# Motives of Children for Digital Gaming and Physical Activity and Their Parents’ Perceptions: Cross-Sectional Matched-Pair Study

**DOI:** 10.2196/80129

**Published:** 2026-03-02

**Authors:** Felix Wachholz, Nicole Gamper, Sarah Kruse, Ruben Maria Anderlan, Martin Schnitzer

**Affiliations:** 1 Department of Sport Science, Faculty for Psychology and Sport Science, Universität Innsbruck, Fürstenweg 185, Innsbruck, 6020, Austria, 43 512 507 ext 45857

**Keywords:** video gaming, digital gaming, physical exercise, parental, leisure time activities, adolescence

## Abstract

**Background:**

Physical activity (PA) is essential for the healthy development of children. However, the pervasive presence of digital technologies has made digital gaming (DG) a prominent part of children’s everyday lives. As children grow up immersed in these digital environments, concerns about reduced PA have intensified. Given that adults, particularly parents and guardians, play a central role in guiding children’s behavior, their understanding of children’s motivational drivers for both PA and DG is of particular relevance.

**Objective:**

This study aimed to explore the motivational differences underlying children’s engagement in either PA or DG. Specifically, the study investigated five distinct motivational scales (recreation, social interaction, coping, competition, and skill) to determine which motives primarily drive behavior in each context. Also, it assessed whether adults accurately perceive these motives in children.

**Methods:**

Data were collected during events using an on-site questionnaire based on the Videogaming Motives Questionnaire. Both children and their accompanying adults completed parallel assessments regarding motives for PA and DG. The final sample included 94 participants forming 49 parent-child pairs. A 3-way mixed ANOVA with group as a between-subjects factor and activity and motive as within-subjects factors was conducted to examine group, activity, and motive effects and their interactions. To further explore these effects, a series of 2 × 5 repeated measures ANOVAs were conducted to examine the interaction between activity type and motivational dimension across groups, followed by separate multivariate tests per motive.

**Results:**

A significant interaction effect between activity type and motivational dimension emerged in the children’s data (*F*_4,45_=3.93, *P*=.008, partial *η²*=.259). Further analyses showed that motive competition was rated significantly higher for DG than for PA (*F*_1,48_=4.38, *P*=.04, partial *η²*=.084). Among adults, separate multivariate tests for each motivational dimension revealed the largest difference in perceived motive coping (*F*_1,48_=4.72, *P*=.01, partial *η²*=.123), with PA rated higher than DG. Additionally, a significant difference emerged for motive competition (*F*_1,48_=4.10, *P*=.05, partial *η²*=.079), indicating higher ratings for DG compared to PA.

**Conclusions:**

The findings emphasize the complexity of children’s motivational profiles, suggesting that engagement in DG is not necessarily a sign of diminished interest in PA but rather reflects alternative, equally compelling motivations. This nuanced understanding challenges simplistic dichotomies and supports the need for balanced perspectives on children’s activity preferences. Importantly, no statistically significant differences were detected between children’s self-reported motives and adults’ perceptions of their children’s motives, suggesting a general tendency toward similar ratings rather than clear evidence of alignment. These insights can inform the development of more tailored strategies for promoting both physical and digital engagement in a healthy and complementary manner.

## Introduction

### Overview

Children and adolescents are not only the custodians of future societies but also key to achieving sustainable development. Investing in their health, education, and well-being is essential for ensuring economic stability, social cohesion, and environmental resilience in the decades to come [1]. Physical activity (PA) is widely recognized for its numerous health benefits, particularly in childhood and adolescence [2]. Regular engagement in PA has been shown to improve cardiovascular health, enhance cognitive development, and foster social well-being [3,4]. Moreover, recent research further supports the cognitive benefits of PA in children, showing that structured sport-based interventions (eg, decision-making drills) can significantly enhance problem-solving and creative thinking abilities in young athletes [5]. Additionally, participation in sports contributes to lifelong physical literacy, encouraging active lifestyles into adulthood [3,6]. Nowadays, the increasing presence of electronic devices has made them an integral part of our daily lives and especially of children and young people, who grow up surrounded by these “new” technologies [7]. Smartphones, tablets, and computers are commonplace, and most young people use them for a variety of purposes, including digital gaming (DG) [7]. Indeed, large national datasets show that sedentary behavior, which often includes screen time, accounts for a significant proportion (around 50%‐60%) of children and young people’s waking hours [8]. The widespread daily use of electronic devices and regular participation in DG form a crucial context for understanding parental concerns about potential negative effects, such as addiction and reduced PA [9]. Given the significant time invested in sedentary screen-based activities, understanding and promoting engagement in PA is increasingly important for the health and well-being of the young age groups [10,11]. Recent findings among physically active university students demonstrate that even short-term interventions can enhance both physical and cognitive performance, underscoring the enduring interplay between activity, recovery, and cognitive function beyond adolescence [12]. With the rise of digital technologies, children’s leisure activities have evolved, and DG has become an integral part of contemporary youth culture. While DG has often been scrutinized for its association with sedentary behavior, with some research linking excessive DG to negative health outcomes, such as increased obesity risk and decreased PA levels [13], emerging research suggests potential positive cognitive, emotional, and social effects [14-16]. Indeed, Granic et al [14] argue for a more balanced perspective on video games, suggesting that their increasingly complex, varied, realistic, and social nature may offer real-world psychosocial benefits. They summarize research suggesting benefits across cognitive domains, such as improved attention and visual skills, as well as motivational, emotional, and social gains from DG experiences.

Research by Barnett et al [17] highlights a contrast in perspectives between parents and children, particularly regarding active video games (AVGs) and basic movement skills. While parents often held skeptical views, perceiving AVGs as not a substitute for “the real thing” and having limited benefits for skill development or transfer to real life, children held a more expansive view of reality and reported using AVGs as a learning tool, perceiving skill acquisition as highly transferable [17], especially in the context of alterations needed in society over time [18]. This difference in perception of rather active digital games, wherein parents have expressed concerns regarding screen time and sedentary behavior [13] and have delineated a distinction between the “virtual” and “real” world [17], contrasts with children’s perspective of DG as a meaningful social space that fosters autonomy and connection [19]. Considering the increasing complexity and interactivity of modern video games, research needs to move beyond simply labeling them as “good” or “bad” and develop equally complex models to understand their influence on players and which underlying motives contribute to their universal appeal and encourage participation of the young population.

### Conflict of Leisure Time—Motivations for PA and Gaming

Understanding what drives engagement in both PA and DG is increasingly pertinent in contemporary society. Gaming in a digital form has become a dominant form of leisure behavior among youth, raising concerns about its potential to displace time that could otherwise be spent on PA. A study by Salmensalo et al [20] found that Finnish vocational students who primarily used PCs for DG were more than twice as likely to exhibit insufficient levels of moderate-to-vigorous PA, suggesting that the DG context and platform may play a role in reinforcing inactivity. Although total DG time was not significantly associated with physical inactivity, the results suggest a conflict hypothesis, which could state that time spent on digital entertainment can compete with PA pursuits. This may be particularly relevant for younger populations, such as children, who face a limited amount of discretionary leisure time. As digital games continue to evolve in complexity and immersive potential, children may increasingly be drawn to screen-based engagement at the expense of PA, creating a behavioral tension between immediate digital gratification and the long-term health benefits of active lifestyles. Related to PA, the primary motivations encompass a range of factors, including the pursuit of improved physical and psychological well-being [21,22], the experience of enjoyment and pleasure [23,24], and the desire for social interaction and support [21] or competition [25]. These motivations can vary depending on individual characteristics such as age and gender [15,21,26,27], as well as the activity undertaken [23].

Similarly, the motivations for engaging in DG are multifaceted, extending beyond mere entertainment or recreation to include the satisfaction of psychological needs such as competence, autonomy, and socialization [15]. Individuals are often driven by the sense of achievement within games [17], the opportunities for social connection and competition [28], and the potential of immersion and escapism into virtual worlds [17]. To comprehensively assess these multifaceted motivations behind DG [15], instruments like the Videogaming Motives Questionnaire (VMQ) have been developed [29] to identify and measure the various underlying motives for playing digital games. An examination of these underlying motivations is critical for designing effective implications to promote healthy behaviors and for better understanding the profound influence of digital entertainment in our lives [23].

### Divergences in Perceptions

A substantial point of divergence exists in the value and purpose attributed to nonactive DG by parents compared to children [7,9,17]. Parents often hold largely negative perceptions of DG, with concerns frequently centering around violent content and the potential for addiction. Further evidence indicates that evening blue light exposure can negatively affect sleep quality, motor function, and cognitive performance in young individuals, highlighting the potential risks of excessive screen use for children’s overall well-being [30], which might be seen negatively by parents as well. This aversion can be attributed, at least in part, to what has been termed a “generational difference,” which hinders parents’ ability to relate to DG [9].

This perspective is in contrast to the multifaceted motivations that children have for engaging with video games. As stated, children engage in DG for the sake of enjoyment, stress reduction, social interaction, the management of boredom, and competition [31]. Martucci et al [15] further delineate a comprehensive array of DG motivations, encompassing recreation, social interaction, coping, violent reward, fantasy, cognitive development, customization, and competition. Moreover, initial findings suggest that children perceive DG as more recreational and skill-enhancing than their parents do [32]. While parents may primarily focus on the perceived negative impacts of DG, existing research highlights potential benefits of DG that parents might overlook [14,33,34]. These include improvements in cognitive skills, motivation, emotion regulation, and social domains. For instance, certain games have been shown to enhance attention and spatial reasoning skills, promote persistence, provide a secure environment for managing negative emotions, and facilitate social interaction [14,35]. Rhodes et al [36] also address a related concern, highlighting that parents perceive screen time, whether active or sedentary, as a potential substitute for other activities they deem more valuable, such as homework, socializing, and sleeping. This observation suggests a more extensive set of concerns among parents regarding the opportunity cost of time allocated to DG.

In contrast, Ng et al [37] found that for children who play sports video games, it can be an important part of their overall “Physical Activity Relationships,” potentially linking to their interest in watching sports and participating in PA, for example, children who actively play soccer are also attracted to soccer simulations like EA Sports’ EA FC [38]. This finding suggests that some children may perceive a connection between the virtual world of sports-gaming and the real world of sports, a link that parents may not fully recognize or value. Although previous studies have examined differences in perceptions between parents and children in the contexts of DG and PA, these domains have largely been investigated in isolation. For example, Franzò et al [39] identified a discrepancy between how parents and children perceive the emotional impact of DG, with parents expressing more concern and children reporting a more nuanced view. Similarly, Kutner et al [40] found that while children view DG as a socially meaningful activity, parents tend to emphasize its potential negative effects, such as interference with academic performance and reduced socialization. In the realm of PA, Tay et al [41] explored children’s motivations for engaging in PA, noting that parental support and access are crucial factors. Ostermeier et al [42] further highlighted parental concerns about decreased PA levels during the COVID-19 pandemic and the importance of peer influence in children’s reengagement with active routines. Despite the value of these findings, there remains a notable gap in the literature: to date and to the best of our knowledge, no study has systematically investigated how parent-child perception differences operate simultaneously across both DG and PA domains. Given the increasing relevance of both behaviors in youth development and health, integrative studies bridging these perspectives are warranted [42].

While parents often perceive DG as excessive, violent, or potentially addictive, and thus harmful to their child’s well-being [9], children are more likely to see it as a meaningful part of their social world, offering opportunities for autonomy, social connection, and identity development [19].

In conclusion, the perceptions of DG and PA might differ significantly between parents and children. While parents often express concern about DG, focusing on its potential negative impacts, lack of “real-world” value compared to PA, and a generational gap in understanding—children tend to be motivated by fun, social interaction, and skill development. They may also perceive a stronger connection between virtual and real-world PAs. We hypothesize that children are expected to show similar motivational patterns for PA and DG, with only minor differences across specific motives, as the structure of the motives for both activities appears to be similar. Moreover, we hypothesize that parents will perceive higher motivational relevance for PA compared to DG in their children, particularly in dimensions related to PA or social engagement. Lastly, certain motivational dimensions—such as coping or competition—will be rated significantly higher for DG than for PA by children. This study seeks to investigate these divergent perceptions by examining whether parents underestimate the potential positive aspects of DG and how children view both DG and PA as integral parts of their daily lives. By exploring these possible perception discrepancies, this study aims to contribute to a better understanding of DG’s role in child development. Based on the hypotheses, two research questions (RQs) were formulated.

RQ1: Do children report different motivational patterns for engaging in PA compared to DG, and which motivational dimensions are more strongly associated with each activity type?

RQ2: To what extent do parents’ perceptions of their children’s motives for PA and DG align with the children’s self-reported motives?

## Methods

### Recruitment

Participants were recruited at two separate events: the Frühjahrsmesse (spring fair) 2024, held from March 14-17, 2024, in Innsbruck, Austria, and Gamers Heaven, which took place on September 21-22, 2024, in Telfs, Austria. The aim was to collect data from a broad and diverse sample representing Tyrolean society. The Frühjahrsmesse is an established annual fair featuring exhibitors and family-oriented attractions, while Gamers Heaven was held for the first time and specifically targeted individuals interested in DG. In total, 162 participants participated in the questionnaire on-site using a Samsung Galaxy Tab A (T515) for data collection due to its portability. The complete questionnaire is available in Multimedia Appendix 1 (original-German) and Multimedia Appendix 2 (translated-English), and the item-level statistics are provided in Multimedia Appendix 3. After excluding incomplete or unmatched responses, the final analytical sample comprised 94 participants (49 children and 45 adults), with four adults each participating with two children, resulting in a total of 49 parent-child pairs. The demographic characteristics of the analyzed sample are presented in Table 1. It should be noted that the child sample showed a substantial gender imbalance, with boys comprising the clear majority of participants, which should be considered when interpreting the motivational patterns reported.

**Table 1.** Demographic description of the sample.

	Age (y), mean (SD)	Gender, n (%)	PA^a^>5 h/wk, n (%)	DG^b^>5 h/wk, n (%)
Children (n=49)	12.6 (2.9)	10 (20.4) female	14 (28.6)	32 (65.3)
Adults (n=45)	44.4 (5.7)	25 (55.6) female	8 (17.8)	—^c^

a PA: physical activity.

b DG: digital gaming.

c Not applicable.

To link responses from children and adults, both were instructed to enter a matching code at the beginning of the questionnaire. This code was based on a combination of letters and numbers derived from shared personal information: the first letter of the place of birth, the first and last letters of the mother’s surname, the first letter of the father’s surname, and the sum of birth day and birth month. If an adult accompanied more than one child, their responses were matched with each respective child’s data set. The average time to complete the questionnaire was 23.8 (SD 16.9) minutes.

Since this study aimed to explore motives across both domains, the questionnaire was built upon the VMQ [29], which was adapted for PA. The VMQ is a validated tool for assessing DG motivations. Prior studies have indicated the presence of psychological drivers, such as competition, skill development, and enjoyment, that are relevant to PA and DG activities [43]. This study adapts the VMQ to measure children’s self-reported motivations for PA and DG, as well as their parents’ perceptions, offering a dual-perspective approach to better understand these behavioral patterns. Based on the VMQ, children completed assessments that evaluated 5 psychological scales related to their engagement in PA and DG: recreation, social interaction, coping, competition, and skill. The questionnaire was independently translated from English to German by three researchers. Discrepancies in wording were subsequently discussed collaboratively to reach a consensus, ensuring that the meaning and intent of each question remained as close to the original as possible. Validated for video gaming [29], the questionnaire was modified to PA by replacing the word “video gaming” in the questions with “sport,” as a child-friendly synonym for PA. It should be noted that the term sport was deliberately used in place of PA to ensure conceptual comparability with DG, as both represent structured, goal-directed, and voluntarily chosen activities. Hence, the motive scales were designed to be quasi-identical and were administered separately for PA and DG contexts, respectively, to allow for consistent and comparable measurement across the two domains. Moreover, parents were asked to report their perceptions of their children’s motivations regarding PA and DG, specifically in relation to the underlying motives driving these behaviors. This allowed for comparisons between both children and parents within each activity type and within each group across the two activity types. The questions were presented in a randomized order within the items, with the starting sequence also varied; for example, some participants began with the PA section followed by the DG section, while others completed the sections in the reverse order.

### Ethical Considerations

Prior to participation, written informed consent was obtained from all parents. Participants were informed about the study procedures, the voluntary nature of participation, and their right to withdraw at any time without providing a reason and without any negative consequences. Data were collected anonymously using a tablet-based survey. To allow withdrawal of data after survey completion without recording identifiable information, participants generated a self-created identification code based on predefined rules (eg, initials of parental names and calculated birth date components). This code was not linked to any personal identifiers and could not be traced back to individual participants by the research team. Confidentiality of all participant information was strictly maintained. Participants did not receive any financial or material compensation for their participation. The study was approved by the Board for Ethical Questions in Science at the University of Innsbruck (Certificate 44/2021). All taken procedures were performed in accordance with the Declaration of Helsinki (1964).

### Statistical Analysis

Data were collected from children and their parents, with paired data structures employed to ensure that individual child-parent connections could be accurately matched. This approach allowed for direct comparisons between corresponding participants within each family, facilitating more precise analyses of the relationships between PA and DG behaviors. A 3-way mixed ANOVA was conducted with group (children vs adults) as the between-subjects factor and activity (PA vs DG) and motive (5 motivational dimensions) as within-subjects factors. This design allowed testing for overall group differences, main effects of activity and motive, as well as their 2- and 3-way interactions to examine how motivational patterns varied across activities and age groups.

Moreover, to exploratorily examine differences between children and parents across 5 motivational scales in detail, as well as activity-related differences (PA vs DG) within each group, four separate 2-way repeated measures ANOVAs (2 × 5 design) were conducted. Although a linear mixed-model approach could account for random effects of matched dyads, the ANOVA strategy was chosen due to its interpretability, established robustness with balanced designs, and the primary interest in specific within- and between-group comparisons per motivational scale. The chosen approach allowed for a clear examination of within-subject effects (activity type and scale) and between-group effects (children vs parents), while also enabling follow-up post hoc comparisons. The paired nature of the data was accounted for by conducting repeated measures designs within each group. If Mauchly’s test indicated violations of the sphericity assumption, the Greenhouse-Geisser correction was applied. Moreover, all individual post hoc comparisons were corrected for multiple testing using the Bonferroni procedure, ensuring control of the family-wise error rate. To control for inflation of the Type I error rate due to multiple testing across the four ANOVAs, the α level was adjusted to .0125 using the Bonferroni correction. Partial eta-squared (η²) was reported to quantify effect sizes, and internal consistency of each scale was assessed using Cronbach α. All analyses were performed using SPSS (version 29.0.2.0; IBM Corp).

### Sample Size

To determine the required sample size, an a priori power analysis was conducted using G*Power (version 3.1.9.7) for a repeated measures ANOVA with a within-subjects design [44]. The analysis was based on an expected medium effect size of *f*=0.25, which corresponds to empirical estimates reported by Granic et al [14] video game intervention research, particularly regarding outcomes such as cognitive flexibility, emotion regulation, and persistence. The significance level was set at *α*=.05 and statistical power at 1–*β*=.80. The analysis assumed two measurement points (eg, DG and PA motives) and a correlation among repeated measures of 0.5. Under these parameters, the required sample size was calculated to be 34 matched pairs (ie, 68 individuals) to detect a within-subjects effect with sufficient power.

## Results

### Internal Consistency of Questionnaire

To assess the reliability of the scales used to measure motivational constructs in both PA and DG contexts, Cronbach α coefficients were calculated for each scale. For PA, internal consistency was acceptable to excellent across all scales, with α values as follows: recreation (7 items) *α*=.780, social interaction (11 items) *α*=.910, coping (8 items) *α*=.828, competition (6 items) *α*=.810, and skill (6 items) *α*=.731. Similarly, for the DG context, reliability coefficients indicated strong internal consistency: recreation (7 items) *α*=.841, social interaction (11 items) *α*=.918, coping (8 items) *α*=.867, competition (6 items) *α*=.808, and skill (6 items) *α*=.782. These results suggest that all subscales demonstrate adequate to excellent internal consistency, indicating reliable measurement of the respective motivational dimensions in both domains.

### Comparison of Motives for PA and DG Across Children and Adults

The Greenhouse Geisser-corrected 2 (group: children vs adults) × 2 (activity: PA vs DG) × 5 (motive) mixed ANOVA revealed a significant main effect of motive, *F*_3.416,327.981_=46.93, *P*<.001, partial *η²*=.328, indicating substantial differences across motivational dimensions. The main effect of activity was not significant, *F*_1,96_=0.19, *P*=.66, partial *η²*=.002, suggesting similar overall motivation levels between PA and DG.

Importantly, a significant activity × motive interaction emerged, *F*_3.474,333.540_=7.48, *P*<.001, partial *η²*=.072, indicating that differences in motivation between PA and DG varied depending on the specific motive. Post hoc pairwise comparisons confirmed that motives differed significantly from one another (all *P*<.001), with recreation and competition being rated higher than coping, social, and skill motives overall.

Neither the main effect of group (*F*_1,96_=0.07, *P*=.79, partial *η²*=.001) nor any interaction involving group reached statistical significance. Thus, motivational profiles were broadly similar between children and adults, both in overall intensity and in how they differentiated between PA and DG motives.

To examine the robustness of the findings across recruitment sites (Frühjahrsmesse and Gamers Heaven), separate 2 (group: child vs adult) × 2 (activity: PA vs DG) × 5 (motive) mixed ANOVAs were conducted for each location [45]. The analyses revealed highly consistent patterns, with significant main effects of motive at both sites (all *P*<.01) and no significant main or interaction effects involving group. The activity × motive interaction was significant at the Frühjahrsmesse but not at Gamers Heaven. Overall, the motivational structure was comparable across sites, confirming the robustness of the main results. In sum, across both age groups, participants distinguished between motivational dimensions, and these motives varied between PA and DG. However, children and adults showed comparable patterns. Because the 3-way mixed ANOVA did not yield any interactions involving the group factor, the subsequent analyses were conducted solely to provide a more detailed description of the within-group motivational patterns for PA and DG. For this purpose, separate 2 × 5 mixed ANOVAs were calculated for children and adults. These follow-up analyses are descriptive in nature and are intended to illustrate group-specific profiles. All inferential conclusions are based on the results of the abovementioned 3-way model.

### Comparison of Motives for PA and DG Among Children

Multivariate tests revealed a significant main effect of motives, *F*_4,45_=34.34, *P*<.001, partial *η²*=.753, indicating substantial differences across motivational dimensions. The main effect of activity was not significant between PA and DG. However, a significant interaction between motives and activity emerged, *F*_4,45_=3.93, *P*=.008, partial *η²*=.259, indicating that the difference in motives ratings between PA and DG varied depending on the motive. The repeated measures ANOVA confirmed the significant main effect of motive, *F*_3.43,164.57_=24.41, *P*<.001, partial *η²*=.337. An almost significant motive × activity interaction was also observed, *F*_3.43,164.57_=3.43, *P*=.02, partial *η²*=.067. The main effect of activity remained nonsignificant, *P*=.78, across all correction methods.

**Figure 1.**
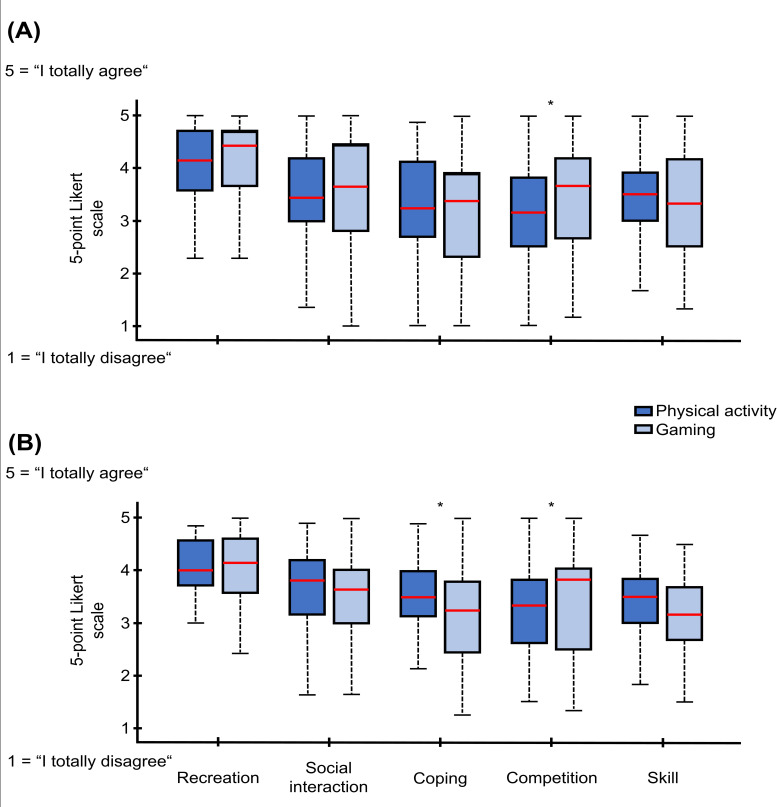
Differences between physical activity (dark blue) and digital gaming (light blue) in relation to the motives recreation, social interaction, coping, competition, and skill are displayed. (A) The differences of children are presented. (B) The results of the adults. Answers to the questions of each motive were given on a 5-point Likert scale. The asterisks (*) indicate significant differences of the post hoc results, which failed to reach significance after Bonferroni correction.

Simple multivariate tests conducted for each motive revealed that only competition showed a significant group difference, *F*_1,48_=4.38, *P*=.04, partial *η²*=.084, as children rated DG as significantly more competition-oriented than PA. The results are demonstrated in Figure 1A.

### Comparison of Motives for PA and DG Among Adults

For adults, multivariate tests revealed a significant main effect of motives, *F*_4,45_=20.68, *P*<.001, partial *η²*=.648, indicating substantial differences across motivational dimensions. The main effect of activity was not significant, *F*_1,48_=1.40, *P*=.24, partial *η²*=.028. Importantly, the interaction between motive and activity was significant, *F*_4,45_=6.23, *P*<.001, partial *η²*=.356. This suggests that the difference in motivation between PA and DG depends on the specific motive being assessed. The within-subject tests confirmed the main effect of motive, *F*_3.06,146.71_=23.36, *P*<.001, partial *η²*=.327. The interaction effect was also significant, *F*_3.37,161.71_=4.78, *P*=.002, partial *η²*=.090, indicating that motives varied across domains between PA and DG. The main effect of activity remained nonsignificant (*P*=.24). Separate multivariate tests for each individual scale showed that the largest difference for adults between PA and DG occurred in perceived coping, *F*_1,48_=4.72, *P*=.01, partial *η²*=.123. Additionally, a significant difference was found for perceived competition, *F*_1,48_=4.10, *P*=.05, partial *η²*=.079. Other scales did not show significant group differences (all *P*.15). The results are displayed in Figure 1B.

### Comparison of PA Motives Between Children and Adults

**Figure 2.**
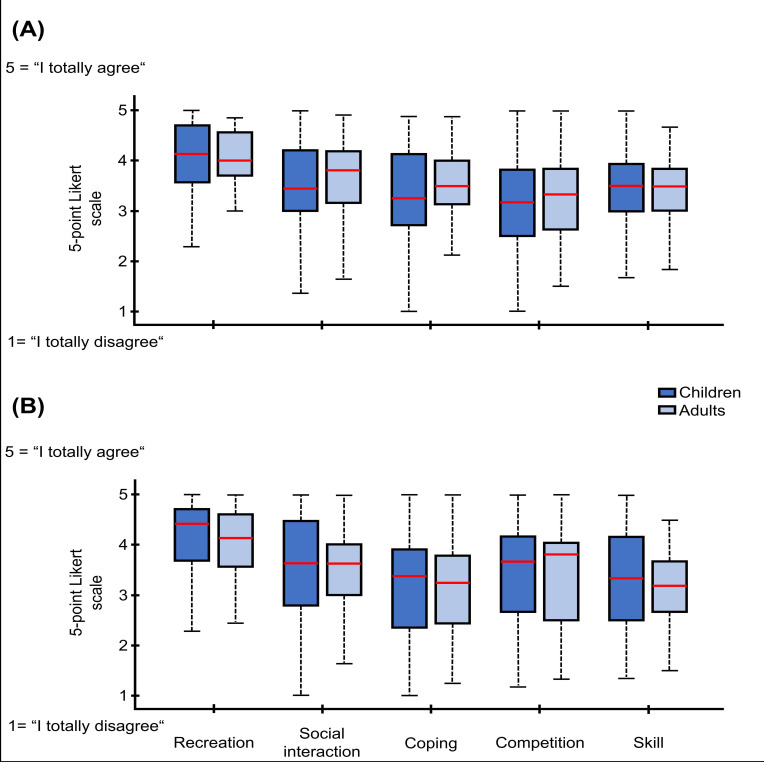
Variances between children (dark blue) and adults (light blue) in relation to the motives recreation, social interaction, coping, competition, and skill are demonstrated. (A) The differences related to PA are presented. (B) The results of DG. Answers to the questions of each motive could be given on a 5-point Likert scale.

Related to PA, multivariate tests revealed a significant main effect of motives, *F*_4,45_=29.19, *P*<.001, partial *η²*=.722, indicating substantial differences in motives across the five domains. In contrast, the main effect of group was not significant, *F*_1,48_=0.21, *P*=.65, partial *η²*=.004. The motive × group interaction also did not reach statistical significance, *F*_4,45_=1.97, *P*=.12, partial *η²*=.149. To further explore potential group differences on individual motivational scales, multivariate tests were conducted separately for each scale. None of the 5 motivational dimensions showed statistically significant differences between children and adults. A visualization of the results is shown in Figure 2A.

### Comparison of DG Motives Between Children and Adults

By investigating whether motivational profiles differ between children and adults across DG, a significant multivariate main effect of motive was observed, *F*_4,45_=43.16, *P*<.001, partial *η²*=.793, indicating substantial differences across motivational dimensions. The main effect of group was not significant, *F*_1,48_=0.95, *P*=.334, partial *η²*=.019. Moreover, the interaction between motive and group also failed to reach significance, *F*_4,45_=0.32, *P*=.88, partial *η²*=.026. Separate multivariate tests were conducted to explore potential differences between activity types for each motivational scale individually. None of the 5 scales reached statistical significance. The results are demonstrated in Figure 2B.

## Discussion

### Principal Results

The present study aimed to explore motivational differences in children’s engagement in PA versus DG and the extent to which parents accurately perceive these motivations. Drawing on 5 distinct motivational dimensions—recreation, social interaction, coping, competition, and skill—we addressed two research questions concerning motivational patterns, intergenerational perception alignment, and differential associations of specific motives with activity type.

A notable and unexpected finding of the present study was that the analyses did not detect evidence of discrepancies between children’s self-reported motives and their parents’ perceptions, despite prior literature suggesting meaningful gaps between self- and proxy-reports [7,9,17]. In contrast to this established work, our results showed no statistically reliable divergence between self- and parent-reported motives across activity domains. Importantly, this pattern should not be interpreted as evidence of true parent-child alignment. Following the principles of null hypothesis significance testing, the finding represents an absence of statistical evidence for a difference—not confirmation of equivalence in motivational understanding.

To examine whether this result might reflect a sampling artifact (eg, parents at a gaming-focused event like Gamers Heaven being more attuned to DG), we conducted sensitivity analyses [45] separately for the Frühjahrsmesse and Gamers Heaven subsamples. The motivational patterns were highly consistent across locations: both subsamples showed strong main effects of motive and, critically, no group-related main or interaction effects. The activity × motive interaction presented to be significant at the Frühjahrsmesse but not at Gamers Heaven, which is likely attributable to reduced statistical power due to the much smaller number of matched pairs at the latter site. However, an equally plausible interpretation is that the gaming-affine Gamers Heaven sample may differ systematically from the spring fair sample in ways that influence motivational patterns. In general, this consistency indicates that the absence of parent-child differences should not be attributed solely to recruitment-site characteristics or increased parental familiarity with DG. Nonetheless, several contextual factors may have contributed to the lack of detectable divergence, including shared situational cues during data collection, relatively high parental involvement in the activities assessed, and the inherently observable nature of motives such as recreation or competition. At the same time, subtle differences may exist but remain undetected due to measurement limitations, shared method variance, or limited statistical power for small effects [46]. These considerations emphasize the need for cautious interpretation: the present results indicate that no discrepancies were detected within this sample, but they should not be taken as evidence of robust parent-child congruence in motivational perceptions.

### Motivational Aspects of PA and DG

Regarding RQ1, if children report different motivational patterns for engaging in PA compared to DG, the results indicate that children do not report systematically different motivational patterns across the 5 dimensions when comparing PA to DG. However, some tendencies emerged that warrant attention. In particular, competition was rated more strongly as a motive in relation to DG than PA, suggesting it may play a distinct role in driving DG activities. This suggests that the motive of competition—known to be relevant in both PA [25] and DG [28]—may be more strongly fulfilled in DG contexts than in PA settings for children. A possible underlying explanation is the accessibility of modern networked play against human opponents, as seen in formats such as Battle Royale games (eg, “Fortnite”) [47] or competitive modes like the Weekend League in “EA FC” [48]. While attending local area network parties back in the days used to require considerable effort, including transporting DG hardware to physical venues, today’s competitive DG experiences can be accessed conveniently from home. While the relatively small sample size may limit the generalizability of the findings, the presence of statistically significant effects even under conservative testing procedures indicates that the observed differences are unlikely to result from random variation alone. Thus, although caution is warranted in interpreting marginal effects or visual trends, the detected significant results can be considered meaningful within the scope of the present design and sample characteristics.

Interestingly, the majority of motivational means of children were in general slightly higher for DG across the board, except for motives skill (Figure 1A) and recreation (Figure 2A), which showed reverse patterns. The gaming industry designs games to be highly engaging and, arguably, even addictive [48-50], potentially addressing psychological motives such as recreation and coping. However, while game mechanics can be programmed, genuine social interaction cannot—yet it showed a higher mean value, albeit without reaching statistical significance. The ability to communicate with peers in real time while playing may further enhance the appeal of DG by strengthening social motives. In summary, games have evolved rapidly in recent years, offering a wide variety of experiences that fulfill a range of important psychological needs. Notably, recreation, interpreted as a general intrinsic or personally valued motivation, consistently received the highest ratings across both activities and groups. This supports the notion of a possible “leisure time conflict,” where both active and passive behaviors are motivated by similarly strong internal drivers, potentially competing for the child’s limited free time [20]. These findings point to the complexity of children’s motivational structures and emphasize that DG as an activity may not necessarily arise from a lack of motivation for PA but from competing, equally compelling motives.

Finally, we examined whether certain motivational dimensions were more strongly associated with either activity. Again, the motive competition emerged as the most activity-specific, being more strongly endorsed for DG by both children and parents. This aligns with prior research suggesting that DG behavior is often motivated by comfort, ease, or relaxation [51,52]. The convergence between generational views on competition adds confidence to this interpretation and underscores its relevance as a potential target for interventions aiming to reduce DG time. Other motives showed relatively balanced activation across PA and DG, further supporting the idea of overlapping and competing motivational structures in leisure contexts. The findings highlight the potentially overlapping nature of children’s motivations for PA versus DG activity. The general alignment between children’s self-perceptions and their parents’ beliefs is promising for family-based intervention approaches. Importantly, the consistently high endorsement of intrinsic motivations like recreation across both activity types reinforces the idea that children are not necessarily passive recipients of screen-based content but are actively motivated by various factors—factors that may also be leveraged to promote PA if carefully aligned.

Focusing on RQ2, to what extent do parents perceive their children’s motives for PA and DG correctly, the analysis did not detect statistically significant differences between children’s self-reported motives and their parents’ perceptions. While this indicates a lack of evidence for systematic discrepancies, it should not be interpreted as evidence of full alignment. The overall pattern suggests that parents’ perceptions broadly resembled children’s self-reports, though the degree of correspondence may vary across motives, which was not anticipated in that form based on existing literature [7,9,17]. We could not find results, which suggests that parents in the tested sample are generally detached from the reasons why children engage in either activity. In the context of PA, parents are generally considered to be fairly accurate in understanding their children’s motives and behaviors [53]. It is plausible that this may also apply to the context of DG, suggesting that parents might be able to perceive and interpret the reasons why their children engage in video games with a comparable level of insight. This could be particularly true for more observable or socially shared motives, such as competition or social interaction, which are easier for parents to recognize. However, some nonsignificant trends pointed to potential perceptual differences. Specifically, the motives of social interaction, coping, and skill were perceived by parents as more relevant for PA than indicated by the children’s self-reports, although these differences did not reach statistical significance. This trend may reflect generational assumptions or aspirational biases—parents might attribute more socially or developmentally valued motives like social interaction or skill development to active behavior [9,54]. Although not statistically robust, these trends merit further exploration, especially considering their potential impact on parental support for children’s activities and a better understanding of parents related to DG nowadays.

### Limitations

Several limitations should be acknowledged. First, although the VMQ [29] was originally validated for video gaming contexts, it was applied to both DG and PA settings in this study. This decision was made as the questionnaire closely aligned with the research questions, and the included motives overlapped substantially with those known to be relevant in PA. It should be noted that the term “sport” was used instead of “physical activity” to ensure conceptual comparability with DG, as both represent structured and intentionally chosen activities. However, this substitution narrows the construct to organized or leisure sport and does not capture the full range of everyday PA (eg, active play, active transport, unstructured movement). As such, the present findings primarily reflect motives for sport participation rather than PA in a broader behavioral sense. Future studies should therefore complement this approach by assessing motivation across the full spectrum of children’s PA behaviors. Moreover, the motivational scales used in this study were not validated against objective behavioral measures. Thus, while the results reflect participants’ self-reported motives, future research should examine how these relate to actual behavioral engagement in PA and DG. However, future research should aim to validate the VMQ across both contexts. Second, while the sample size was relatively modest, it closely matched the a priori power analysis, which indicated that 68 participants would be sufficient to detect moderate effects. The use of ANOVA and the observed moderate effect sizes (eg, partial *η²*) further support the robustness of the findings, especially after Bonferroni-corrected alpha level. Third, a potential sampling bias cannot be entirely ruled out, as data were also collected at the “Gamers Heaven” event, which is an event for analog and DG-affine people. However, efforts were made to minimize bias by approaching a broad and casual family audience directly on site. Finally, the study was limited to the region of Tyrol, which may constrain the generalizability of the results to other populations. However, it presents a first matched-data approach showing the potential competing motives related to PA and DG of children. Lastly, the gender distribution differed between groups, with a lower proportion of female participants among children compared to adults. This imbalance should be considered when interpreting the results, as gender-related differences may have influenced motivational patterns.

### Conclusions

These findings highlight the nuanced and multifaceted nature of children’s motivational structures. Rather than indicating a lack of interest in PA—or in this study, sport—DG may emerge as a response to equally compelling, but distinct, motivational drivers. This complexity underscores the importance of further investigating these trends—particularly in relation to how they shape parental support for different types of activities. A deeper understanding of contemporary DG motives may also enhance parents’ ability to relate to and support their children’s engagement in both digital and physical pursuits.

## Supplementary material

10.2196/80129 Multimedia Appendix 1 Original questionnaire.

10.2196/80129 Multimedia Appendix 2 Translated questionnaire.

10.2196/80129 Multimedia Appendix 3 Item-level scale statistics.

## Abbreviations

AVG: active video games
DG: digital gaming
PA: physical activity
VMQ: Videogaming Motives Questionnaire
