# Supplementary material for: Motives of Children for Digital Gaming and Physical Activity and Their Parents’ Perceptions: Cross-Sectional Matched-Pair Study
Source: JMIR Pediatr Parent. 2026 Mar 2;9:e80129. doi: 10.2196/80129 (PMC12954707; doi:10.2196/80129)
Supplement: Multimedia Appendix 1 [file pediatrics-v9-e80129-s001.docx]

Fragebogen für die Frühjahresmesse 2024

Gaming vs. Sport

Beginn des Blocks: Einleitung

Q50 Sehr geehrte Messebesucherin, sehr geehrter Messebesucher, vielen Dank für das Interesse an unsere Umfrage! Im Folgenden werden Fragen zu Motiven und Verhaltensweisen gestellt. Es ist wichtig, dass du/Sie wahrheitsgetreu antwortest/antworten, es gibt keine falschen oder richtigen Antworten. Die Daten werden absolut vertraulich behandelt und lassen keine Rückschlüsse auf dich/Sie als Person zu. Wenn du/Sie unten auf "Ich stimme zu" klickst/klicken, stimmst du/stimmen Sie der Teilnahme an einer wissenschaftlichen Studie zu, für die die erhobenen Daten verwendet werden. Das ändert nichts daran, dass die Daten weder an Dritte weitergegeben, noch in irgendeiner Form anderweitig verwendet werden. Dadurch wird eingewilligt, dass die Teilnahme freiwillig erfolgt und bekannt ist, dass jederzeit, auch ohne Angabe von Gründen, die Bearbeitung der Online-Studie abgebrochen werden kann, ohne dass daraus Nachteile irgendwelcher Art entstehen.

Q52 Die neue Datenschutzgrundverordnung ermöglicht, dass die erhobenen Daten auf Wunsch zu einem späteren Zeitpunkt eingesehen oder wieder aus dem Datensatz entnommen werden können. Dazu wird ein individualisierter Code benötigt. Wählen Sie als Kind und Erziehungsberechtigte/r bitte einen Code gemeinsam (am besten die Vorgehensweise unten auf den Erwachsenen von Ihnen anwenden). Dadurch können wir keinen Rückschluss auf Euch als Personen ziehen, aber Eure Daten matchen, was für die Studie wichtig ist. Bitte erstellen Sie das Codewort nach den angegebenen Regeln: erster Buchstabe Ihres Geburtsortes (z.B. **I**nnsbruck → **I**) erster Buchstabe des Vornamens Ihrer Mutter (z.B. **A**nnemarie → **A**) letzter Buchstabe des Vornamens Ihrer Mutter (z.B. Annemari**e** → **E**) erster Buchstabe des Vornamens Ihres Vaters (z.B. **T**homas → **T**) Summe Ihres Geburtstages und Geburtsmonats (z.B. **17**.**11**.1995 → **17** + **11** = 28 oder z.B. **03**.**05**.1996 → **03** + **05** = 08) Also z.B. **IAET28** bzw. **IAET08**

________________________________________________________________

Q51 Sollten Fragen auftauchen steht der Studienleiter Dr. Felix Wachholz per E-Mail (felix.wachholz@uibk.ac.at) zur Verfügung. Abermals vielen herzlichen Dank und noch einen schönen Tag auf der Messe!

Ende des Blocks: Einleitung

Beginn des Blocks: Kind oder Erwachsener

Q1 Bist du/Sind Sie Kind oder Erwachsener?

- Kind (1)
- Erwachsener (2)

Ende des Blocks: Kind oder Erwachsener

Beginn des Blocks: Demographische Daten - Kind

Diese Frage anzeigen:

If Bist du/Sind Sie Kind oder Erwachsener? = Kind

Q2 Welchem Geschlecht fühlst du dich zugehörig?

- Männlich (1)
- Weiblich (2)
- Nichtbinär/drittes Geschlecht (3)
- Keine Angabe (4)

Diese Frage anzeigen:

If Bist du/Sind Sie Kind oder Erwachsener? = Kind

Q3 Wie alt bist du?

|  | 6 | 7 | 8 | 9 | 10 | 11 | 12 | 13 | 14 | 15 | 16 | 17 |
| --- | --- | --- | --- | --- | --- | --- | --- | --- | --- | --- | --- | --- |

| Alter () | 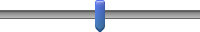 |
| --- | --- |

Diese Frage anzeigen:

If Bist du/Sind Sie Kind oder Erwachsener? = Kind

| 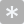 |
| --- |

Q4 Wo wohnst du derzeit (gib bitte deine Postleitzahl ein)?

________________________________________________________________

Ende des Blocks: Demographische Daten - Kind

Beginn des Blocks: Sport - Kind

Q9 Wie viel Zeit verbringst du durchschnittlich pro Woche mit moderater bis intensiver körperlicher Aktivität?

- Weniger als 1 Stunde pro Woche (1)
- 1 - 2,5 Stunden pro Woche (2)
- mehr als 2,5 - 5 Stunden pro Woche (3)
- mehr als 5 Stunden pro Woche (4)

Q21 Bist du Mitglied in einem Sportverein?

- Ja (1)
- Nein (2)

Q22 Welchen Sport betreibst du hauptsächlich?

- Fußball (1)
- Tennis (2)
- Klettern (3)
- Kraftsport (4)
- Handball (5)
- Basketball (6)
- Wintersport (Skifahren, Snowboarden, Rodeln...) (7)
- Sonstige (8) __________________________________________________
- Gar keinen (9)

Überspringen bis: Q27 Wenn Welchen Sport betreibst du hauptsächlich? = Gar keinen

Q23 Würdest du gerne Profisportler werden?

- Ja (1)
- Eher ja (2)
- Eher nein (3)
- Nein (4)

| Seitenumbruch |  |
| --- | --- |


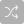


|  |
| --- |

| Q24 Was sind deine Motive für das Sporttreiben? | stimme überhaupt nicht zu (1) | stimme eher nicht zu (2) | weder noch (3) | stimme eher zu (4) | stimme absolut zu (5) |
| --- | --- | --- | --- | --- | --- |
| ich Sport machen genieße (1) |  |  |  |  |  |
| Sport machen mich unterhält (2) |  |  |  |  |  |
| ich Spaß habe (3) |  |  |  |  |  |
| es unterhaltsam ist (4) |  |  |  |  |  |
| ich den Sport mag (5) |  |  |  |  |  |
| es mich entspannt (6) |  |  |  |  |  |
| es mir hilft Stress lozuwerden (7) |  |  |  |  |  |
| ich neue Freunde gewinne (8) |  |  |  |  |  |
| es mir ermöglicht neue Menschen kennenzulernen (9) |  |  |  |  |  |
| ich mit meinen Freunden durch Sport in Kontakt bleibe (10) |  |  |  |  |  |
| ich es genieße in der Gruppe Sport zu machen (11) |  |  |  |  |  |
| ich es mag mit anderen Sport zu machen (12) |  |  |  |  |  |
| ich dadurch in eine Gruppe gehöre, die ich mag (13) |  |  |  |  |  |
| andere mich schätzen, wenn ich mitspiele (14) |  |  |  |  |  |
| ich mich mit meinen Freunden über den Sport unterhalten kann (15) |  |  |  |  |  |
| ich aufgrund meiner Leistung im Sport Anerkennung bekomme (16) |  |  |  |  |  |
| die anderen mich mögen, wenn ich mitspiele (17) |  |  |  |  |  |
| alle meine Freunde Sport machen (18) |  |  |  |  |  |
| es mir hilft meine täglichen Probleme zu vergessen (19) |  |  |  |  |  |
| ich meine Sorgen vergesse (20) |  |  |  |  |  |
| es mir erlaubt aus der Welt zu fliehen (21) |  |  |  |  |  |
| Sport mir ermöglicht, mich besser zu fühlen, wenn ich frustriert bin (22) |  |  |  |  |  |
| Sport machen meine Stimmung verbessert (23) |  |  |  |  |  |
| es mir hilft negative Energie freizulassen (24) |  |  |  |  |  |
| ich mich dadurch nicht ausgegrenzt fühle (25) |  |  |  |  |  |
| wenn ich wütend oder verärgert über jemanden bin, ich durch Sport eine Auseinandersetzung mit dieser Person vermeide (26) |  |  |  |  |  |
| es mir hilft meine Aggressivität zu bündeln (27) |  |  |  |  |  |
| Sport machen mein Adrenalinlevel erhöht (28) |  |  |  |  |  |
| Sport meine Emotionen stimuliert (29) |  |  |  |  |  |
| Sport machen aufregend ist (30) |  |  |  |  |  |
| ich gerne andere Sportler besiege (31) |  |  |  |  |  |
| ich es mag zu gewinnen (32) |  |  |  |  |  |
| ich gerne beweise, dass ich besser als andere bin (33) |  |  |  |  |  |
| ich es mag andere Sportler zu provozieren (34) |  |  |  |  |  |
| ich mich in dem Sport leistungsfähig fühle (35) |  |  |  |  |  |
| ich es genieße, mich mit anderen zu messen (36) |  |  |  |  |  |
| das Sport machen eine geistige Herausforderung darstellt (37) |  |  |  |  |  |
| das Sport treiben mich schlauer macht (38) |  |  |  |  |  |
| das Sport machen mich zum Nachdenken bringt (39) |  |  |  |  |  |
| das Sport machen meine Sinne schärft (40) |  |  |  |  |  |
| das Sport machen mich reizt (41) |  |  |  |  |  |
| das Sport machen meine Fähigkeiten verbessert (42) |  |  |  |  |  |

| Seitenumbruch |  |
| --- | --- |

Q27 Wie viele Stunden am Tag verbringst du durchschnittlich mit Sitzen?

________________________________________________________________

Q28 Wie viele Stunden schläfst du durchschnittlich pro Nacht?

________________________________________________________________

Q29 Wie würdest du die Qualität deines Schlafes bewerten?

- Sehr gut (1)
- Ziemlich gut (2)
- Weder noch (3)
- Ziemlich schlecht (4)
- Sehr schlecht (5)

Ende des Blocks: Sport - Kind

Beginn des Blocks: Mediennutzung - Kind

Q10 Wie würdest du dich selbst in Bezug auf Gaming/E-Sports einstufen?

- Professioneller Spieler: Ich verdiene regelmäßig bedeutende Einnahmen aus E-Sport (Preisgelder, Sponsoren, Gehalt von Clubs). (1)
- Amateur: Ich spiele E-Sport, verdiene jedoch kein signifikantes Einkommen. (2)
- Regelmäßiger Spieler: Ich spiele Videospiele oder E-Sport mehrmals pro Woche, nehme aber nicht an offiziellen Turnieren und Ligen teil. (3)
- Gelegentlicher Spieler: Ich spiele Videospiele oder E-Sport mehrmals im Monat oder seltener, und nehme nicht an offiziellen Turnieren und Ligen teil. (4)
- Nichtspieler: Ich spiele keine Videospiele oder E-Sport. (5)

Überspringen bis: Q19 Wenn Wie würdest du dich selbst in Bezug auf Gaming/E-Sports einstufen? = Nichtspieler: Ich spiele keine Videospiele oder E-Sport.

Q13 Würdest du gerne professioneller E-Sportler werden?

- Ja (1)
- Eher ja (2)
- Eher nein (3)
- Nein (4)

Q14 Bist du Mitglied in einem E-Sports bzw. Gaming Verein?

- Ja (1)
- Nein (2)

Q15 Seit wie vielen Jahren spielst du Videospiele?

- Seit weniger als 12 Monaten (1)
- Seit 2 - 3 Jahren (2)
- Seit mehr als 3 - 5 Jahren (3)
- Seit über 5 Jahren (4)

| Seitenumbruch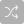 |  |
| --- | --- |

| Q16 Was sind deine Motive für das Gaming/E-Sport? | stimme überhaupt nicht zu (1) | stimme eher nicht zu (2) | weder noch (3) | stimme eher zu (4) | stimme absolut zu (5) |
| --- | --- | --- | --- | --- | --- |
| ich Videospiele spielen genieße (1) |  |  |  |  |  |
| die Videospiele mich unterhalten (2) |  |  |  |  |  |
| ich Spaß habe (3) |  |  |  |  |  |
| es unterhaltsam ist (4) |  |  |  |  |  |
| ich die Videospiele mag (5) |  |  |  |  |  |
| es mich entspannt (6) |  |  |  |  |  |
| es mir hilft Stress loszuwerden (7) |  |  |  |  |  |
| ich neue Freunde gewinne (8) |  |  |  |  |  |
| es mir ermöglicht neue Menschen kennenzulernen (9) |  |  |  |  |  |
| ich mit meinen Freunden durch das Videospielen in Kontakt bleibe (10) |  |  |  |  |  |
| ich es genieße in der Gruppe Videospiele zu spielen (11) |  |  |  |  |  |
| ich es mag, mit anderen online oder im gleichen Raum zu spielen (12) |  |  |  |  |  |
| ich dadurch in eine Gruppe gehöre, die ich mag (13) |  |  |  |  |  |
| andere mich schätzen, wenn ich mitspiele (14) |  |  |  |  |  |
| ich mich mit meinen Freunden über die Videospiele unterhalten kann (15) |  |  |  |  |  |
| ich aufgrund meiner Spielerfolge Anerkennung bekomme (16) |  |  |  |  |  |
| die anderen mich mögen, wenn ich mitspiele (17) |  |  |  |  |  |
| alle meine Freunde spielen (18) |  |  |  |  |  |
| es mir hilft meine täglichen Probleme zu vergessen (19) |  |  |  |  |  |
| ich meine Sorgen vergesse (20) |  |  |  |  |  |
| es mir erlaubt aus der realen Welt zu fliehen (21) |  |  |  |  |  |
| Videospielen mir ermöglicht, mich besser zu fühlen, wenn ich frustriert bin (22) |  |  |  |  |  |
| Videospielen meine Stimmung verbessert (23) |  |  |  |  |  |
| es mir hilft negative Energie freizulassen (24) |  |  |  |  |  |
| ich mich dadurch nicht ausgegrenzt fühle (25) |  |  |  |  |  |
| wenn ich wütend oder verärgert über jemanden bin, ich durch das Videospielen eine Auseinandersetzung mit dieser Person vermeide (26) |  |  |  |  |  |
| es mir hilft meine Aggressivität zu bündeln (27) |  |  |  |  |  |
| Videospiele mein Adrenalinlevel erhöhen (28) |  |  |  |  |  |
| ich in eine fantastische/fiktive Welt eintauchen kann (29) |  |  |  |  |  |
| es mir gefällt, mich als Teil einer Geschichte zu fühlen (30) |  |  |  |  |  |
| es mir Spaß macht, mich in jedem Videospiel in einen neuen Charakter hineinzuversetzen (31) |  |  |  |  |  |
| ich mich in dem Videospiel als jemand besonderes fühle (32) |  |  |  |  |  |
| ich es mag, die Welt zu erkunden und neue Dinge zu entdecken (33) |  |  |  |  |  |
| Videospiele meine Emotionen stimulieren (34) |  |  |  |  |  |
| Videospiele aufregend sind (35) |  |  |  |  |  |
| ich es mag, Dinge in Videospielen zu personalisieren (36) |  |  |  |  |  |
| ich gerne Dinge in Videospielen baue, zum Beispiel Häuser oder andere Konstruktionen (37) |  |  |  |  |  |
| ich gerne meine eigene Welt in Videospielen erstelle (38) |  |  |  |  |  |
| ich in Videospielen gerne verschiedene Elemente verwende, um neue Dinge zu schaffen (39) |  |  |  |  |  |
| ich es mag, das Aussehen meiner Charaktere zu entwerfen oder zu verändern (40) |  |  |  |  |  |
| ich gerne andere Videospieler besiege (41) |  |  |  |  |  |
| ich es mag zu gewinnen (42) |  |  |  |  |  |
| ich gerne beweise, dass ich besser als andere bin (43) |  |  |  |  |  |
| ich es mag andere Videospieler zu provozieren (44) |  |  |  |  |  |
| ich mich in dem Videospiel leistungsfähig fühle (45) |  |  |  |  |  |
| ich es genieße, mich mit anderen zu messen (46) |  |  |  |  |  |
| die Videospiele eine geistige Herausforderung darstellen (47) |  |  |  |  |  |
| die Videospiele mich schlauer machen (48) |  |  |  |  |  |
| die Videospiele mich zum Nachdenken bringen (49) |  |  |  |  |  |
| die Videospiele meine Sinne schärfen (50) |  |  |  |  |  |
| die Videospiele mich reizen (51) |  |  |  |  |  |
| die Videospiele meine Fähigkeiten verbessern (52) |  |  |  |  |  |

| Seitenumbruch |  |
| --- | --- |

Q17 Wie viel Zeit verbringst du durchschnittlich pro Woche mit Gaming/E-Sport?

- Weniger als 5 Stunden pro Woche (1)
- 5 - 10 Stunden pro Woche (2)
- 11 - 20 Stunden pro Woche (3)
- 21 - 30 Stunden pro Woche (4)
- Mehr als 30 Stunden pro Woche (5)

Q18 Welches Genre von Videospielen spielst du vorwiegend?

- First-Person-Shooter (z.B. Counter-Strike, Halo, Quake, Unreal Tournament, Overwatch, Paladins, Fortnite, PlayerUnknown's Battlegrounds, Rainbow Six Siege, Call of Duty, Battlefield) (1)
- Echtzeit-Strategiespiele (z.B. Dota 2, Empire Earth, Homeworld, Sudden Strike, Warcraft) (2)
- Sport- und Rennsimulationen (z.B. FIFA, Driver San Francisco, RIDE 2, Wreckfest, The Crew) (3)
- Mobile Games (4)
- Sonstige (5) __________________________________________________

Q19 Wie viele Stunden benutzt du durchschnittlich das Internet pro Tag?

- Nie (1)
- Weniger als 1 Stunde (2)
- 1 - 3 Stunden (3)
- mehr als 3 - 5 Stunden (4)
- Mehr als 5 Stunden (5)

Q20 Wie viele Stunden benutzt du durchschnittlich dein Smartphone (Internetzugang, Möglichkeit Spiele zu spielen) pro Tag?

- Ich habe kein eigenes Smartphone (1)
- Weniger als 1 Stunde (2)
- 1 - 3 Stunden (3)
- mehr als 3 - 5 Stunden (4)
- Mehr als 5 Stunden (5)

Ende des Blocks: Mediennutzung - Kind

Beginn des Blocks: Wahrnehmung Verhalten - Kind

Q30 Wie würdest du dein Verhalten in den folgenden Bereichen einschätzen?

|  | zu wenig (1) | ziemlich wenig (2) | angemessen (3) | ziemlich viel (4) | zu viel (5) |
| --- | --- | --- | --- | --- | --- |
| Tägliche Videospieldauer (1) |  |  |  |  |  |
| Tägliche Internetnutzung (2) |  |  |  |  |  |
| Durchschnittlich moderate sportliche Aktivität pro Tag (3) |  |  |  |  |  |
| Durchschnittliche tägliche Sitzzeiten (4) |  |  |  |  |  |

Q47 Wie würdest du deinen Gesundheitszustand bewerten?

|  | Schlecht (1) | Nicht so gut (2) | Gut (3) | Sehr gut (4) | Ausgezeichnet (5) |
| --- | --- | --- | --- | --- | --- |
| Gesundheitszustand (1) |  |  |  |  |  |

Ende des Blocks: Wahrnehmung Verhalten - Kind

Beginn des Blocks: Demographische Daten - Erwachsen

Diese Frage anzeigen:

If Bist du/Sind Sie Kind oder Erwachsener? = Erwachsener

Q5 Welchem Geschlecht fühlen Sie sich zugehörig?

- Männlich (1)
- Weiblich (2)
- Nichtbinär/drittes Geschlecht (3)
- Keine Angabe (4)

Diese Frage anzeigen:

If Bist du/Sind Sie Kind oder Erwachsener? = Erwachsener

| 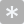 |
| --- |

Q6 Wie alt sind Sie?

________________________________________________________________

Diese Frage anzeigen:

If Bist du/Sind Sie Kind oder Erwachsener? = Erwachsener

| 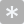 |
| --- |

Q7 Wo wohnen Sie derzeit (geben Sie bitte Ihre Postleitzahl ein)?

________________________________________________________________

Diese Frage anzeigen:

If Bist du/Sind Sie Kind oder Erwachsener? = Erwachsener

Q8 Was ist Ihr derzeitiger Beschäftigungsstatus?

- Selbstständig (1)
- Freiberufler:in (2)
- Angestellt, Vollzeitbeschäftigung (3)
- Angestellt, Teilzeitbeschäftigung (4)
- Geringfügige Beschäftigung (5)
- In Ausbildung (6)
- Ohne Beschäftigung, arbeitssuchend (7)
- Arbeitsunfähig (8)
- Pensioniert (9)
- Sonstige (10) __________________________________________________
- Keine Angabe (11)

Ende des Blocks: Demographische Daten - Erwachsen

Beginn des Blocks: Sport - Erwachsen

Q36 Wie viel Zeit verbringen Sie durchschnittlich pro Woche mit moderater bis intensiver körperlicher Aktivität?

- Weniger als 1 Stunde pro Woche (1)
- 1 - 3 Stunden pro Woche (2)
- mehr als 3 - 6 Stunden pro Woche (3)
- Mehr als 6 Stunden (4)

Q37 Wie viel Zeit verbringt Ihr Kind/Ihre Kinder durchschnittlich pro Woche mit moderater bis intensiver körperlicher Aktivität?

- weniger als 1 Stunde pro Woche (1)
- 1 - 3 Stunden pro Woche (2)
- mehr als 3 - 6 Stunden pro Woche (3)
- mehr als 6 Stunden pro Woche (4)

Q38 Sind Sie Mitglied in einem Sportverein?

- Ja (1)
- Nein (2)

| Seitenumbruch |  |
| --- | --- |


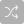


|  |
| --- |

| Q33 Was denken Sie, sind die Motive Ihres Kindes/Ihrer Kinder Sport zu machen? | stimme überhaupt nicht zu (1) | stimme eher nicht zu (2) | weder noch (3) | stimme eher zu (4) | stimme absolut zu (5) |
| --- | --- | --- | --- | --- | --- |
| es Sport machen genießt (1) |  |  |  |  |  |
| es durch Sport machen unterhalten wird (2) |  |  |  |  |  |
| es Spaß hat (3) |  |  |  |  |  |
| es unterhaltsam ist (4) |  |  |  |  |  |
| es den Sport mag (5) |  |  |  |  |  |
| es mein Kind entspannt (6) |  |  |  |  |  |
| es ihr/ihm hilft Stress loszuwerden (7) |  |  |  |  |  |
| es neue Freunde gewinnt (8) |  |  |  |  |  |
| es ihr/ihm ermöglicht neue Menschen kennenzulernen (9) |  |  |  |  |  |
| es mit ihren/seinen Freunden durch Sport in Kontakt bleibt (10) |  |  |  |  |  |
| es genießt in der Gruppe Sport zu machen (11) |  |  |  |  |  |
| es mag, mit anderen Sport zu machen (12) |  |  |  |  |  |
| es dadurch in eine Gruppe gehört, die es mag (13) |  |  |  |  |  |
| es von anderen geschätzt wird, wenn es mitspielt (14) |  |  |  |  |  |
| es sich mit ihren/seinen Freunden über den Sport unterhalten kann (15) |  |  |  |  |  |
| es aufgrund ihrer/seiner Leistung im Sport Anerkennung bekommt (16) |  |  |  |  |  |
| die anderen es mögen, wenn es mitspielt (17) |  |  |  |  |  |
| alle ihre/seine Freunde Sport machen (18) |  |  |  |  |  |
| es hilft ihre/seine täglichen Probleme zu vergessen (19) |  |  |  |  |  |
| sie ihre/er seine Sorgen vergisst (20) |  |  |  |  |  |
| es ihr/ihm erlaubt aus der Welt zu fliehen (21) |  |  |  |  |  |
| Sport ihr/ihm ermöglicht, sich besser zu fühlen, wenn es frustriert ist (22) |  |  |  |  |  |
| Sport machen ihre/seine Stimmung verbessert (23) |  |  |  |  |  |
| es ihr/ihm hilft negative Energie freizulassen (24) |  |  |  |  |  |
| es sich dadurch nicht ausgegrenzt fühlt (25) |  |  |  |  |  |
| wenn es wütend oder verärgert über jemanden ist, es durch Sport eine Auseinandersetzung mit dieser Person vermeidet (26) |  |  |  |  |  |
| es ihr/ihm hilft Aggressivität zu bündeln (27) |  |  |  |  |  |
| Sport machen ihren/seinen Adrenalinlevel erhöht (28) |  |  |  |  |  |
| Sport ihre/seine Emotionen stimuliert (29) |  |  |  |  |  |
| Sport machen aufregend ist (30) |  |  |  |  |  |
| es gerne andere Sportler besiegt (31) |  |  |  |  |  |
| es mag zu gewinnen (32) |  |  |  |  |  |
| es gerne beweist, dass es besser als andere ist (33) |  |  |  |  |  |
| es mag andere Sportler zu provozieren (34) |  |  |  |  |  |
| es sich in dem Sport leistungsfähig fühlt (35) |  |  |  |  |  |
| es genießt, sich mit anderen zu messen (36) |  |  |  |  |  |
| das Sport machen eine geistige Herausforderung darstellt (37) |  |  |  |  |  |
| das Sport treiben es schlauer macht (38) |  |  |  |  |  |
| das Sport machen es zum Nachdenken bringt (39) |  |  |  |  |  |
| das Sport machen ihre/seine Sinne schärft (40) |  |  |  |  |  |
| das Sport machen es reizt (41) |  |  |  |  |  |
| das Sport machen ihre/seine Fähigkeiten verbessert (42) |  |  |  |  |  |

| Seitenumbruch |  |
| --- | --- |

Q39 Wie viele Stunden am Tag verbringen Sie durchschnittlich mit Sitzen?

________________________________________________________________

Q40 Wie viele Stunden schlafen Sie durchschnittlich pro Nacht?

________________________________________________________________

Q41 Wie viele Stunden schläft Ihr Kind/Ihre Kinder durchschnittlich pro Nacht?

________________________________________________________________

Q42 Wie würden Sie die Qualität Ihres Schlafes bewerten?

- Sehr gut (1)
- Ziemlich gut (2)
- Weder noch (3)
- Ziemlich schlecht (4)
- Sehr schlecht (5)

Ende des Blocks: Sport - Erwachsen

Beginn des Blocks: Mediennutzung - Erwachsen

Q31 Was glauben Sie, wie würde sich Ihr Kind selbst in Bezug auf E-Sport einstufen?

- Professioneller Spieler: Ihr Kind verdient regelmäßig bedeutende Einnahmen aus E-Sport (Preisgelder, Sponsoren, Gehalt von Clubs). (1)
- Amateur: Ihr Kind spielt E-Sport, verdient jedoch kein signifikantes Einkommen. (2)
- Regelmäßiger Spieler: Ihr Kind spielt Videospiele oder E-Sport mehrmals pro Woche, nimmt aber nicht an offiziellen Turnieren und Ligen teil. (3)
- Gelegentlicher Spieler: Ihr Kind spielt Videospiele oder E-Sport mehrmals im Monat oder seltener, Ihr Kind nimmt nicht an offiziellen Turnieren und Ligen teil. (4)
- Nichtspieler: Ihr Kind spielt keine Videospiele oder E-Sport. (5)

Überspringen bis: Q34 Wenn Was glauben Sie, wie würde sich Ihr Kind selbst in Bezug auf E-Sport einstufen? = Nichtspieler: Ihr Kind spielt keine Videospiele oder E-Sport.

Q32 Glauben Sie Ihr Kind würde gerne E-Sport Profi (Professioneller Spieler) werden?

- Ja (1)
- Eher ja (2)
- Eher nein (3)
- Nein (4)

Q45 Wissen Sie, welche Spiele Ihr Kind/Ihre Kinder spielen?

- Ja (1)
- Eher ja (2)
- Eher nein (3)
- Nein (4)

| Seitenumbruch |  |
| --- | --- |


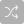


| Q26 Was denken Sie, sind die Motive für das Gaming/E-Sport Ihres Kindes/Ihrer Kinder? | stimme überhaupt nicht zu (1) | stimme eher nicht zu (2) | weder noch (3) | stimme eher zu (4) | stimme absolut zu (5) |
| --- | --- | --- | --- | --- | --- |
| es Videospiele spielen genießt (1) |  |  |  |  |  |
| es durch die Videospiele unterhalten wird (2) |  |  |  |  |  |
| es Spaß hat (3) |  |  |  |  |  |
| es unterhaltsam ist (4) |  |  |  |  |  |
| es die Videospiele mag (5) |  |  |  |  |  |
| es mein Kind entspannt (6) |  |  |  |  |  |
| es ihr/ihm hilft Stress loszuwerden (7) |  |  |  |  |  |
| es neue Freunde gewinnt (8) |  |  |  |  |  |
| es ihr/ihm ermöglicht neue Menschen kennenzulernen (9) |  |  |  |  |  |
| es mit ihren/seinen Freunden durch das Videospielen in Kontakt bleibt (10) |  |  |  |  |  |
| es genießt in der Gruppe Videospiele zu spielen (11) |  |  |  |  |  |
| es mag mit anderen online oder im gleichen Raum zu spielen (12) |  |  |  |  |  |
| es dadurch in eine Gruppe gehört, die es mag (13) |  |  |  |  |  |
| es von anderen geschätzt wird, wenn es mitspielt (14) |  |  |  |  |  |
| es sich mit ihren/seinen Freunden über die Videospiele unterhalten kann (15) |  |  |  |  |  |
| es von anderen für ihre/seine Spielerfolge Anerkennung bekommt (16) |  |  |  |  |  |
| die anderen es mögen, wenn es mitspielt (17) |  |  |  |  |  |
| alle ihre/seine Freunde spielen (18) |  |  |  |  |  |
| es ihr/ihm hilft, die täglichen Probleme zu vergessen (19) |  |  |  |  |  |
| es ihre/seine Sorgen vergisst (20) |  |  |  |  |  |
| es ihr/ihm erlaubt aus der realen Welt zu fliehen (21) |  |  |  |  |  |
| Videospielen ihr/ihm ermöglicht, sich besser zu fühlen, wenn es frustriert ist (22) |  |  |  |  |  |
| Videospielen ihre/seine Stimmung verbessert (23) |  |  |  |  |  |
| es ihr/ihm hilft negative Energie freizulassen (24) |  |  |  |  |  |
| es sich dadurch nicht ausgegrenzt fühlt (25) |  |  |  |  |  |
| wenn es wütend oder verärgert über jemanden ist, es durch das Videospielen eine Auseinandersetzung mit dieser Person vermeidet (26) |  |  |  |  |  |
| es ihr/ihm hilft Aggressivität zu bündeln (27) |  |  |  |  |  |
| Videospiele ihr/sein Adrenalinlevel erhöhen (28) |  |  |  |  |  |
| es in eine fantastische/fiktive Welt eintauchen kann (29) |  |  |  |  |  |
| es ihr/ihm gefällt, sich als Teil einer Geschichte zu fühlen (30) |  |  |  |  |  |
| es ihr/ihm Spaß macht, sich in jedem Videospiel in einen neuen Charakter hineinzuversetzen (31) |  |  |  |  |  |
| es sich in dem Videospiel als jemand besonderes fühlt (32) |  |  |  |  |  |
| es genießt, die Welt zu erkunden und neue Dinge zu entdecken (33) |  |  |  |  |  |
| Videospiele ihre/seine Emotionen stimulieren (34) |  |  |  |  |  |
| Videospiele aufregend sind (35) |  |  |  |  |  |
| es genießt, Dinge in Videospielen zu personalisieren (36) |  |  |  |  |  |
| es gerne Dinge in Videospielen baut, zum Beispiel Häuser oder andere Konstruktionen (37) |  |  |  |  |  |
| es gerne ihre/seine eigene Welt in Videopielen erstellt (38) |  |  |  |  |  |
| es in Videospielen gerne verschiedene Elemente verwendet, um neue Dinge zu schaffen (39) |  |  |  |  |  |
| es mag, das Aussehen ihrer/seiner Charaktere zu entwerfen oder zu verändern (40) |  |  |  |  |  |
| es gerne andere Videospieler besiegt (41) |  |  |  |  |  |
| es mag zu gewinnen (42) |  |  |  |  |  |
| es gerne beweist, dass es besser als andere ist (43) |  |  |  |  |  |
| es mag andere Videospieler zu provozieren (44) |  |  |  |  |  |
| es sich in dem Videospiel leistungsfähig fühlt (45) |  |  |  |  |  |
| es genießt, sich mit anderen zu messen (46) |  |  |  |  |  |
| die Videospiele eine geistige Herausforderung darstellen (47) |  |  |  |  |  |
| die Videospiele es schlauer machen (48) |  |  |  |  |  |
| die Videospiele es zum Nachdenken bringen (49) |  |  |  |  |  |
| die Videospiele ihre/seine Sinne schärfen (50) |  |  |  |  |  |
| die Videospiele es reizen (51) |  |  |  |  |  |
| die Videospiele ihre/seine Fähigkeiten verbessern (52) |  |  |  |  |  |

| Seitenumbruch |  |
| --- | --- |

Q34 Wie viele Stunden schauen Sie durchschnittlich TV am Tag?

- Weniger als 1 Stunde (1)
- 1 - 3 Stunden (2)
- mehr als 3 - 5 Stunden (3)
- Mehr als 5 Stunden (4)

Q35 Wie viele Stunden benutzen Sie durchschnittlich das Internet pro Tag?

- Weniger als 1 Stunde (1)
- 1 - 3 Stunden (2)
- mehr als 3 - 5 Stunden (3)
- Mehr als 5 Stunden (4)

Ende des Blocks: Mediennutzung - Erwachsen

Beginn des Blocks: Wahrnehmung Verhalten - Erwachsen

Q43 Wie würden Sie Ihr persönliches Verhalten in den folgenden Bereichen einschätzen?

|  | zu wenig (1) | ziemlich wenig (2) | angemessen (3) | ziemlich viel (4) | zu viel (5) |
| --- | --- | --- | --- | --- | --- |
| Tägliche Videospieldauer (1) |  |  |  |  |  |
| Tägliche Internetnutzung (2) |  |  |  |  |  |
| Durchschnittliche moderate Aktivität pro Tag (3) |  |  |  |  |  |
| Durchschnittliche tägliche Sitzzeiten (4) |  |  |  |  |  |

Q48 Wie würden Sie Ihren Gesundheitszustand bewerten?

|  | Schlecht (1) | Nicht so gut (2) | Gut (3) | Sehr gut (4) | Ausgezeichnet (5) |
| --- | --- | --- | --- | --- | --- |
| Gesundheitszustand (1) |  |  |  |  |  |

Q44 Wie nehmen Sie folgende Bereiche bei Ihrem Kind/Ihren Kindern wahr?

|  | zu wenig (1) | ziemlich wenig (2) | angemessen (3) | ziemlich viel (4) | zu viel (5) |
| --- | --- | --- | --- | --- | --- |
| Tägliche Spieldauer (1) |  |  |  |  |  |
| Tägliche Internetnutzung (2) |  |  |  |  |  |
| Durchschnittliche moderate Aktivität pro Tag (3) |  |  |  |  |  |
| Durchschnittliche tägliche Sitzzeiten (4) |  |  |  |  |  |

Q49 Wie würden Sie den Gesundheitszustand Ihres Kindes bewerten?

|  | Schlecht (1) | Nicht so gut (2) | Gut (3) | Sehr gut (4) | Ausgezeichnet (5) |
| --- | --- | --- | --- | --- | --- |
| Gesundheitszustand (1) |  |  |  |  |  |

Ende des Blocks: Wahrnehmung Verhalten - Erwachsen

Beginn des Blocks: Messe

Q46 Welche Verbesserungen und/der Erweiterungen würdest du dir/würden Sie sich für den Gaming- & E-Sport-Bereich in Tirol wünschen?

________________________________________________________________

Q47 Welche Gaming- & E-Sport-Angebote würdest du dir/würden Sie sich generell in Tirol wünschen?

________________________________________________________________

Q48 Welche Kanäle nutzt du/nutzen Sie aktuell im Gaming Bereich? (Facebook, Instagram, TikTok, YouTube, Twitch.tv, Discord, WhatsApp, o.Ä.)

________________________________________________________________

Q49 Wie zufrieden warst du/waren Sie mit der Frühjarsmesse 2024? Bitte im Schnulnotensystem bewerten (1 = sehr gut ; 6 = ungenügend)

|  | 1 | 2 | 3 | 4 | 5 | 6 |
| --- | --- | --- | --- | --- | --- | --- |

| Die Frühjahrsmesse 2024 erhält von mir dir Note () | 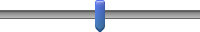 |
| --- | --- |
| Der Gaming-Bereich auf der Frühjahrsmesse 2024 erhält die Note () | 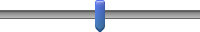 |

Ende des Blocks: Messe
