# Supplementary material for: Motives of Children for Digital Gaming and Physical Activity and Their Parents’ Perceptions: Cross-Sectional Matched-Pair Study
Source: JMIR Pediatr Parent. 2026 Mar 2;9:e80129. doi: 10.2196/80129 (PMC12954707; doi:10.2196/80129)
Supplement: Multimedia Appendix 2 [file pediatrics-v9-e80129-s002.docx]

Questionnaire for the Frühjahresmesse 2024 – translated using LLM based on GPT 4o

Gaming vs. Sport

**Start of Block: Introduction**

**Q50** Dear Frühjahresmesse 2024 visitor,
Thank you for your interest in our survey! The following questions will address motives and behaviors. It is important that you answer truthfully, as there are no right or wrong answers. The data will be treated with absolute confidentiality and will not allow any conclusions to be drawn about you as a person. By clicking "I agree" below, you consent to participate in a scientific study for which the collected data will be used. This does not change the fact that the data will neither be shared with third parties nor used in any other way. By agreeing, you confirm that your participation is voluntary and that you are aware that you can terminate the online study at any time, even without providing a reason, without any disadvantages of any kind.

**Q52** The new General Data Protection Regulation allows you to view or have your data removed from the dataset at a later time upon request. For this, an individualized code is required. If you are a child and a legal guardian, please create a code together (it is best to apply the procedure below to the adult among you). This ensures that we cannot identify you as individuals, but we can match your data, which is important for the study.
Please create the codeword according to the following rules:

- First letter of your place of birth (e.g., Innsbruck → I)
- First letter of your mother's first name (e.g., Annemarie → A)
- Last letter of your mother's first name (e.g., Annemarie → E)
- First letter of your father's first name (e.g., Thomas → T)
- Sum of your birth day and birth month (e.g., 17.11.1995 → 17 + 11 = 28 or e.g., 03.05.1996 → 03 + 05 = 08)

For example: IAET28 or IAET08.

**Q51** If you have any questions, the study leader Dr. Felix Wachholz can be contacted via email ([felix.wachholz@uibk.ac.at](mailto:felix.wachholz@uibk.ac.at)).
Once again, thank you very much, and have a great day at the trade fair!

**End of Block: Introduction**

**Start of Block: Child or Adult**

**Q1** Are you a child or an adult?

- Child (1)
- Adult (2)

**End of Block: Child or Adult**

**Start of Block: Demographic Data - Child**
*This question is displayed if "Are you a child or an adult?" = Child.*

**Q2** What gender do you identify with?

- Male (1)
- Female (2)
- Non-binary/third gender (3)
- Prefer not to say (4)

*This question is displayed if "Are you a child or an adult?" = Child.*

**Q3** How old are you?
6 7 8 9 10 11 12 13 14 15 16 17

Age: ______

*This question is displayed if "Are you a child or an adult?" = Child.*

**Q4** Where do you currently live (please enter your postal code)?

**End of Block: Demographic Data - Child**

**Start of Block: Sports - Child**

**Q9** How much time do you spend on average per week engaging in moderate to intense physical activity?

- Less than 1 hour per week (1)
- 1 - 2.5 hours per week (2)
- More than 2.5 - 5 hours per week (3)
- More than 5 hours per week (4)

**Q21** Are you a member of a sports club?

- Yes (1)
- No (2)

**Q22** What sport do you primarily engage in?

- Soccer (1)
- Tennis (2)
- Climbing (3)
- Strength training (4)
- Handball (5)
- Basketball (6)
- Winter sports (skiing, snowboarding, sledding, etc.) (7)
- Other (8) __________________________________________________
- None (9)

*Skip to Q27 if "What sport do you primarily engage in?" = None.*

**Q23** Would you like to become a professional athlete?

- Yes (1)
- Rather yes (2)
- Rather no (3)
- No (4)

**Q24** What are your motives for engaging in sports?
*Rate the following statements:*

- Strongly disagree (1)
- Rather disagree (2)
- Neither agree nor disagree (3)
- Rather agree (4)
- Strongly agree (5)

| **Statement** | **1** | **2** | **3** | **4** | **5** |
| --- | --- | --- | --- | --- | --- |
| I enjoy doing sports. | o | o | o | o | o |
| Sports entertain me. | o | o | o | o | o |
| I have fun. | o | o | o | o | o |
| It is entertaining. | o | o | o | o | o |
| I like the sport. | o | o | o | o | o |
| It relaxes me. | o | o | o | o | o |
| It helps me relieve stress. | o | o | o | o | o |
| I make new friends. | o | o | o | o | o |
| It allows me to meet new people. | o | o | o | o | o |
| I stay in touch with my friends through sports. | o | o | o | o | o |
| I enjoy doing sports in a group. | o | o | o | o | o |
| I like doing sports with others. | o | o | o | o | o |
| It makes me feel part of a group I like. | o | o | o | o | o |
| Others appreciate me when I participate. | o | o | o | o | o |
| I can talk to my friends about sports. | o | o | o | o | o |
| I receive recognition for my performance in sports. | o | o | o | o | o |
| Others like me when I participate. | o | o | o | o | o |
| All my friends do sports. | o | o | o | o | o |
| It helps me forget my daily problems. | o | o | o | o | o |
| I forget my worries. | o | o | o | o | o |
| It allows me to escape from the world. | o | o | o | o | o |
| Sports help me feel better when I am frustrated. | o | o | o | o | o |
| Doing sports improves my mood. | o | o | o | o | o |
| It helps me release negative energy. | o | o | o | o | o |
| I don’t feel excluded because of it. | o | o | o | o | o |
| When I am angry or upset with someone, sports help me avoid a confrontation. | o | o | o | o | o |
| It helps me channel my aggression. | o | o | o | o | o |
| Doing sports increases my adrenaline level. | o | o | o | o | o |
| Sports stimulate my emotions. | o | o | o | o | o |
| Doing sports is exciting. | o | o | o | o | o |
| I like defeating other athletes. | o | o | o | o | o |
| I like winning. | o | o | o | o | o |
| I like proving that I am better than others. | o | o | o | o | o |
| I like provoking other athletes. | o | o | o | o | o |
| I feel capable in sports. | o | o | o | o | o |
| I enjoy competing with others. | o | o | o | o | o |
| Doing sports is a mental challenge. | o | o | o | o | o |
| Doing sports makes me smarter. | o | o | o | o | o |
| Doing sports makes me think. | o | o | o | o | o |
| Doing sports sharpens my senses. | o | o | o | o | o |
| Doing sports excites me. | o | o | o | o | o |
| Doing sports improves my skills. | o | o | o | o | o |

**Q27** How many hours a day do you spend sitting on average?

**Q28** How many hours do you sleep on average per night?

**Q29** How would you rate the quality of your sleep?

- Very good (1)
- Quite good (2)
- Neither good nor bad (3)
- Quite bad (4)
- Very bad (5)

**End of Block: Sports - Child**

**Start of Block: Media Usage - Child**

**Q10** How would you classify yourself in terms of gaming/e-sports?

- Professional player: I regularly earn significant income from e-sports (prize money, sponsorships, club salaries). (1)
- Amateur: I play e-sports but do not earn significant income. (2)
- Regular player: I play video games or e-sports several times a week but do not participate in official tournaments or leagues. (3)
- Occasional player: I play video games or e-sports several times a month or less frequently and do not participate in official tournaments or leagues. (4)
- Non-player: I do not play video games or e-sports. (5)

*Skip to Q19 if "How would you classify yourself in terms of gaming/e-sports?" = Non-player: I do not play video games or e-sports.*

**Q13** Would you like to become a professional e-sports player?

- Yes (1)
- Rather yes (2)
- Rather no (3)
- No (4)

**Q14** Are you a member of an e-sports or gaming club?

- Yes (1)
- No (2)

**Q15** How many years have you been playing video games?

- Less than 12 months (1)
- 2 - 3 years (2)
- More than 3 - 5 years (3)
- Over 5 years (4)

**Q16** What are your motives for gaming/e-sports?
*Rate the following statements:*

- Strongly disagree (1)
- Rather disagree (2)
- Neither agree nor disagree (3)
- Rather agree (4)
- Strongly agree (5)

| **Statement** | **1** | **2** | **3** | **4** | **5** |
| --- | --- | --- | --- | --- | --- |
| I enjoy playing video games. | o | o | o | o | o |
| Video games entertain me. | o | o | o | o | o |
| I have fun. | o | o | o | o | o |
| It is entertaining. | o | o | o | o | o |
| I like video games. | o | o | o | o | o |
| It relaxes me. | o | o | o | o | o |
| It helps me relieve stress. | o | o | o | o | o |
| I make new friends. | o | o | o | o | o |
| It allows me to meet new people. | o | o | o | o | o |
| I stay in touch with my friends through gaming. | o | o | o | o | o |
| I enjoy playing video games in a group. | o | o | o | o | o |
| I like playing with others online or in the same room. | o | o | o | o | o |
| It makes me feel part of a group I like. | o | o | o | o | o |
| Others appreciate me when I play. | o | o | o | o | o |
| I can talk to my friends about video games. | o | o | o | o | o |
| I receive recognition for my gaming achievements. | o | o | o | o | o |
| Others like me when I play. | o | o | o | o | o |
| All my friends play video games. | o | o | o | o | o |
| It helps me forget my daily problems. | o | o | o | o | o |
| I forget my worries. | o | o | o | o | o |
| It allows me to escape from the real world. | o | o | o | o | o |
| Gaming helps me feel better when I am frustrated. | o | o | o | o | o |
| Gaming improves my mood. | o | o | o | o | o |
| It helps me release negative energy. | o | o | o | o | o |
| I don’t feel excluded because of it. | o | o | o | o | o |
| When I am angry or upset with someone, gaming helps me avoid a confrontation. | o | o | o | o | o |
| It helps me channel my aggression. | o | o | o | o | o |
| Video games increase my adrenaline level. | o | o | o | o | o |
| I can immerse myself in a fantastic/fictional world. | o | o | o | o | o |
| I enjoy feeling like part of a story. | o | o | o | o | o |
| I enjoy taking on a new character in every video game. | o | o | o | o | o |
| I feel special in the video game. | o | o | o | o | o |
| I like exploring the world and discovering new things. | o | o | o | o | o |
| Video games stimulate my emotions. | o | o | o | o | o |
| Video games are exciting. | o | o | o | o | o |
| I like personalizing things in video games. | o | o | o | o | o |
| I enjoy building things in video games, such as houses or other constructions. | o | o | o | o | o |
| I like creating my own world in video games. | o | o | o | o | o |
| I enjoy using different elements in video games to create new things. | o | o | o | o | o |
| I like designing or changing the appearance of my characters. | o | o | o | o | o |
| I like defeating other video game players. | o | o | o | o | o |
| I like winning. | o | o | o | o | o |
| I like proving that I am better than others. | o | o | o | o | o |
| I like provoking other video game players. | o | o | o | o | o |
| I feel capable in video games. | o | o | o | o | o |
| I enjoy competing with others. | o | o | o | o | o |
| Video games are a mental challenge. | o | o | o | o | o |
| Video games make me smarter. | o | o | o | o | o |
| Video games make me think. | o | o | o | o | o |
| Video games sharpen my senses. | o | o | o | o | o |
| Video games excite me. | o | o | o | o | o |
| Video games improve my skills. | o | o | o | o | o |

**Q17** How much time do you spend on average per week on gaming/e-sports?

- Less than 5 hours per week (1)
- 5 - 10 hours per week (2)
- 11 - 20 hours per week (3)
- 21 - 30 hours per week (4)
- More than 30 hours per week (5)

**Q18** What genre of video games do you primarily play?

- First-person shooters (e.g., Counter-Strike, Halo, Quake, Unreal Tournament, Overwatch, Paladins, Fortnite, PlayerUnknown's Battlegrounds, Rainbow Six Siege, Call of Duty, Battlefield) (1)
- Real-time strategy games (e.g., Dota 2, Empire Earth, Homeworld, Sudden Strike, Warcraft) (2)
- Sports and racing simulations (e.g., FIFA, Driver San Francisco, RIDE 2, Wreckfest, The Crew) (3)
- Mobile games (4)
- Other (5) __________________________________________________

**Q19** How many hours do you use the internet on average per day?

- Never (1)
- Less than 1 hour (2)
- 1 - 3 hours (3)
- More than 3 - 5 hours (4)
- More than 5 hours (5)

**Q20** How many hours do you use your smartphone (internet access, ability to play games) on average per day?

- I do not own a smartphone (1)
- Less than 1 hour (2)
- 1 - 3 hours (3)
- More than 3 - 5 hours (4)
- More than 5 hours (5)

**End of Block: Media Usage - Child**

**Start of Block: Perception of Behavior - Child**

**Q30** How would you assess your behavior in the following areas?

- Too little (1)
- Quite little (2)
- Adequate (3)
- Quite a lot (4)
- Too much (5)

| **Area** | **1** | **2** | **3** | **4** | **5** |
| --- | --- | --- | --- | --- | --- |
| Daily video game duration | o | o | o | o | o |
| Daily internet usage | o | o | o | o | o |
| Average moderate physical activity per day | o | o | o | o | o |
| Average daily sitting time | o | o | o | o | o |

**Q47** How would you rate your health condition?

- Poor (1)
- Not so good (2)
- Good (3)
- Very good (4)
- Excellent (5)

| **Health condition** | **1** | **2** | **3** | **4** | **5** |
| --- | --- | --- | --- | --- | --- |
| Health condition | o | o | o | o | o |

**End of Block: Perception of Behavior - Child**

**Start of Block: Demographic Data - Adult**
*This question is displayed if "Are you a child or an adult?" = Adult.*

**Q5** What gender do you identify with?

- Male (1)
- Female (2)
- Non-binary/third gender (3)
- Prefer not to say (4)

*This question is displayed if "Are you a child or an adult?" = Adult.*

**Q6** How old are you?

*This question is displayed if "Are you a child or an adult?" = Adult.*

**Q7** Where do you currently live (please enter your postal code)?

*This question is displayed if "Are you a child or an adult?" = Adult.*

**Q8** What is your current employment status?

- Self-employed (1)
- Freelancer (2)
- Employed, full-time (3)
- Employed, part-time (4)
- Marginal employment (5)
- In training (6)
- Unemployed, seeking work (7)
- Unable to work (8)
- Retired (9)
- Other (10) __________________________________________________
- Prefer not to say (11)

**End of Block: Demographic Data - Adult**

**Start of Block: Sports - Adult**

**Q36** How much time do you spend on average per week engaging in moderate to intense physical activity?

- Less than 1 hour per week (1)
- 1 - 3 hours per week (2)
- More than 3 - 6 hours per week (3)
- More than 6 hours (4)

**Q37** How much time does your child/children spend on average per week engaging in moderate to intense physical activity?

- Less than 1 hour per week (1)
- 1 - 3 hours per week (2)
- More than 3 - 6 hours per week (3)
- More than 6 hours per week (4)

**Q38** Are you a member of a sports club?

- Yes (1)
- No (2)

**Q33** What do you think are your child’s/children’s motives for engaging in sports?
*Rate the following statements:*

- Strongly disagree (1)
- Rather disagree (2)
- Neither agree nor disagree (3)
- Rather agree (4)
- Strongly agree (5)

| **Statement** | **1** | **2** | **3** | **4** | **5** |
| --- | --- | --- | --- | --- | --- |
| They enjoy doing sports. | o | o | o | o | o |
| They are entertained by doing sports. | o | o | o | o | o |
| They have fun. | o | o | o | o | o |
| It is entertaining. | o | o | o | o | o |
| They like the sport. | o | o | o | o | o |
| It relaxes them. | o | o | o | o | o |
| It helps them relieve stress. | o | o | o | o | o |
| They make new friends. | o | o | o | o | o |
| It allows them to meet new people. | o | o | o | o | o |
| They stay in touch with their friends through sports. | o | o | o | o | o |
| They enjoy doing sports in a group. | o | o | o | o | o |
| They like doing sports with others. | o | o | o | o | o |
| It makes them feel part of a group they like. | o | o | o | o | o |
| Others appreciate them when they participate. | o | o | o | o | o |
| They can talk to their friends about sports. | o | o | o | o | o |
| They receive recognition for their performance in sports. | o | o | o | o | o |
| Others like them when they participate. | o | o | o | o | o |
| All their friends do sports. | o | o | o | o | o |
| It helps them forget their daily problems. | o | o | o | o | o |
| They forget their worries. | o | o | o | o | o |
| It allows them to escape from the world. | o | o | o | o | o |
| Sports help them feel better when they are frustrated. | o | o | o | o | o |
| Doing sports improves their mood. | o | o | o | o | o |
| It helps them release negative energy. | o | o | o | o | o |
| They don’t feel excluded because of it. | o | o | o | o | o |
| When they are angry or upset with someone, sports help them avoid a confrontation. | o | o | o | o | o |
| It helps them channel their aggression. | o | o | o | o | o |
| Doing sports increases their adrenaline level. | o | o | o | o | o |
| Sports stimulate their emotions. | o | o | o | o | o |
| Doing sports is exciting. | o | o | o | o | o |
| They like defeating other athletes. | o | o | o | o | o |
| They like winning. | o | o | o | o | o |
| They like proving that they are better than others. | o | o | o | o | o |
| They like provoking other athletes. | o | o | o | o | o |
| They feel capable in sports. | o | o | o | o | o |
| They enjoy competing with others. | o | o | o | o | o |
| Doing sports is a mental challenge. | o | o | o | o | o |
| Doing sports makes them smarter. | o | o | o | o | o |
| Doing sports makes them think. | o | o | o | o | o |
| Doing sports sharpens their senses. | o | o | o | o | o |
| Doing sports excites them. | o | o | o | o | o |
| Doing sports improves their skills. | o | o | o | o | o |

**Q39** How many hours per day do you spend sitting on average?

**Q40** How many hours do you sleep on average per night?

**Q41** How many hours does your child/children sleep on average per night?

**Q42** How would you rate the quality of your sleep?

- Very good (1)
- Quite good (2)
- Neither good nor bad (3)
- Quite bad (4)
- Very bad (5)

**End of Block: Sports - Adult**

**Start of Block: Media Usage - Adult**

**Q31** What do you think, how would your child classify themselves in terms of e-sports?

- Professional player: Your child regularly earns significant income from e-sports (prize money, sponsorships, club salaries). (1)
- Amateur: Your child plays e-sports but does not earn significant income. (2)
- Regular player: Your child plays video games or e-sports several times a week but does not participate in official tournaments or leagues. (3)
- Occasional player: Your child plays video games or e-sports several times a month or less frequently and does not participate in official tournaments or leagues. (4)
- Non-player: Your child does not play video games or e-sports. (5)

*Skip to Q34 if "What do you think, how would your child classify themselves in terms of e-sports?" = Non-player: Your child does not play video games or e-sports.*

**Q32** Do you think your child would like to become a professional e-sports player?

- Yes (1)
- Rather yes (2)
- Rather no (3)
- No (4)

**Q45** Do you know which games your child/children play?

- Yes (1)
- Rather yes (2)
- Rather no (3)
- No (4)

**Q26** What do you think are your child’s/children’s motives for gaming/e-sports?
*Rate the following statements:*

- Strongly disagree (1)
- Rather disagree (2)
- Neither agree nor disagree (3)
- Rather agree (4)
- Strongly agree (5)

| **Statement** | **1** | **2** | **3** | **4** | **5** |
| --- | --- | --- | --- | --- | --- |
| They enjoy playing video games. | o | o | o | o | o |
| They are entertained by video games. | o | o | o | o | o |
| They have fun. | o | o | o | o | o |
| It is entertaining. | o | o | o | o | o |
| They like video games. | o | o | o | o | o |
| It relaxes them. | o | o | o | o | o |
| It helps them relieve stress. | o | o | o | o | o |
| They make new friends. | o | o | o | o | o |
| It allows them to meet new people. | o | o | o | o | o |
| They stay in touch with their friends through gaming. | o | o | o | o | o |
| They enjoy playing video games in a group. | o | o | o | o | o |
| They like playing with others online or in the same room. | o | o | o | o | o |
| It makes them feel part of a group they like. | o | o | o | o | o |
| Others appreciate them when they play. | o | o | o | o | o |
| They can talk to their friends about video games. | o | o | o | o | o |
| They receive recognition for their gaming achievements. | o | o | o | o | o |
| Others like them when they play. | o | o | o | o | o |
| All their friends play video games. | o | o | o | o | o |
| It helps them forget their daily problems. | o | o | o | o | o |
| They forget their worries. | o | o | o | o | o |
| It allows them to escape from the real world. | o | o | o | o | o |
| Gaming helps them feel better when they are frustrated. | o | o | o | o | o |
| Gaming improves their mood. | o | o | o | o | o |
| It helps them release negative energy. | o | o | o | o | o |
| They don’t feel excluded because of it. | o | o | o | o | o |
| When they are angry or upset with someone, gaming helps them avoid a confrontation. | o | o | o | o | o |
| It helps them channel their aggression. | o | o | o | o | o |
| Video games increase their adrenaline level. | o | o | o | o | o |
| They can immerse themselves in a fantastic/fictional world. | o | o | o | o | o |
| They enjoy feeling like part of a story. | o | o | o | o | o |
| They enjoy taking on a new character in every video game. | o | o | o | o | o |
| They feel special in the video game. | o | o | o | o | o |
| They enjoy exploring the world and discovering new things. | o | o | o | o | o |
| Video games stimulate their emotions. | o | o | o | o | o |
| Video games are exciting. | o | o | o | o | o |
| They enjoy personalizing things in video games. | o | o | o | o | o |
| They like building things in video games, such as houses or other constructions. | o | o | o | o | o |
| They enjoy creating their own world in video games. | o | o | o | o | o |
| They like using various elements in video games to create new things. | o | o | o | o | o |
| They like designing or changing the appearance of their characters. | o | o | o | o | o |
| They enjoy defeating other video game players. | o | o | o | o | o |
| They like winning. | o | o | o | o | o |
| They like proving that they are better than others. | o | o | o | o | o |
| They like provoking other video game players. | o | o | o | o | o |
| They feel capable in video games. | o | o | o | o | o |
| They enjoy competing with others. | o | o | o | o | o |
| Video games are a mental challenge. | o | o | o | o | o |
| Video games make them smarter. | o | o | o | o | o |
| Video games make them think. | o | o | o | o | o |
| Video games sharpen their senses. | o | o | o | o | o |
| Video games excite them. | o | o | o | o | o |
| Video games improve their skills. | o | o | o | o | o |

**Q34** How many hours do you watch TV on average per day?

- Less than 1 hour (1)
- 1 - 3 hours (2)
- More than 3 - 5 hours (3)
- More than 5 hours (4)

**Q35** How many hours do you use the internet on average per day?

- Less than 1 hour (1)
- 1 - 3 hours (2)
- More than 3 - 5 hours (3)
- More than 5 hours (4)

**End of Block: Media Usage - Adult**

**Start of Block: Perception of Behavior - Adult**

**Q43** How would you assess your personal behavior in the following areas?

- Too little (1)
- Quite little (2)
- Adequate (3)
- Quite a lot (4)
- Too much (5)

| **Area** | **1** | **2** | **3** | **4** | **5** |
| --- | --- | --- | --- | --- | --- |
| Daily video game duration | o | o | o | o | o |
| Daily internet usage | o | o | o | o | o |
| Average moderate activity per day | o | o | o | o | o |
| Average daily sitting time | o | o | o | o | o |

**Q48** How would you rate your health condition?

- Poor (1)
- Not so good (2)
- Good (3)
- Very good (4)
- Excellent (5)

**Q44** How do you perceive the following areas in your child/children?

- Too little (1)
- Quite little (2)
- Adequate (3)
- Quite a lot (4)
- Too much (5)

| **Area** | **1** | **2** | **3** | **4** | **5** |
| --- | --- | --- | --- | --- | --- |
| Daily gaming duration | o | o | o | o | o |
| Daily internet usage | o | o | o | o | o |
| Average moderate activity per day | o | o | o | o | o |
| Average daily sitting time | o | o | o | o | o |

**Q49** How would you rate your child’s health condition?

- Poor (1)
- Not so good (2)
- Good (3)
- Very good (4)
- Excellent (5)

| **Health condition** | **1** | **2** | **3** | **4** | **5** |
| --- | --- | --- | --- | --- | --- |
| Health condition | o | o | o | o | o |

**End of Block: Perception of Behavior - Adult**

**Start of Block: Trade Fair**

**Q46** What improvements and/or expansions would you like to see for the gaming and e-sports sector in Tyrol?

**Q47** What gaming and e-sports offerings would you generally like to see in Tyrol?

**Q48** Which channels do you currently use in the gaming sector? (Facebook, Instagram, TikTok, YouTube, Twitch.tv, Discord, WhatsApp, etc.)

**Q49** How satisfied were you with the Frühjahresmesse 2024? Please rate using the school grading system (1 = very good; 6 = unsatisfactory).

| **Rating** | **1** | **2** | **3** | **4** | **5** | **6** |
| --- | --- | --- | --- | --- | --- | --- |
| The Frühjahresmesse 2024 receives the grade: | () |  |  |  |  |  |
| The gaming section at the Frühjahresmesse 2024 receives the grade: | () |  |  |  |  |  |

**End of Block: Trade Fair**
