# Supplementary material for: Motives of Children for Digital Gaming and Physical Activity and Their Parents’ Perceptions: Cross-Sectional Matched-Pair Study
Source: JMIR Pediatr Parent. 2026 Mar 2;9:e80129. doi: 10.2196/80129 (PMC12954707; doi:10.2196/80129)
Supplement: Multimedia Appendix 3 [file pediatrics-v9-e80129-s003.docx]

**Physical Activity Children**

|  | **Statement** | M | SD | rᵢ₋ₜ | α = Cronbach’s alpha if item deleted |
| --- | --- | --- | --- | --- | --- |
| PA_Recreation1 | I enjoy doing sports. | 4,10 | 1,07 | 0,64 | 0,77 |
| PA_Recreation2 | Sports entertain me. | 4,02 | 1,15 | 0,68 | 0,77 |
| PA_Recreation3 | I have fun. | 4,59 | 0,89 | 0,58 | 0,79 |
| PA_Recreation4 | It is entertaining. | 4,20 | 1,02 | 0,46 | 0,80 |
| PA_Recreation5 | I like the sport. | 4,45 | 0,98 | 0,64 | 0,78 |
| PA_Recreation6 | It relaxes me. | 3,51 | 1,28 | 0,54 | 0,79 |
| PA_Recreation7 | It helps me relieve stress. | 3,65 | 1,41 | 0,42 | 0,82 |
| PA_Social1 | I make new friends. | 3,55 | 1,35 | 0,76 | 0,87 |
| PA_Social2 | It allows me to meet new people. | 3,53 | 1,44 | 0,74 | 0,87 |
| PA_Social3 | I stay in touch with my friends through sports. | 3,53 | 1,44 | 0,61 | 0,88 |
| PA_Social4 | I enjoy doing sports in a group. | 3,76 | 1,36 | 0,68 | 0,88 |
| PA_Social5 | I like doing sports with others. | 4,06 | 1,25 | 0,73 | 0,88 |
| PA_Social6 | It makes me feel part of a group I like. | 3,65 | 1,35 | 0,69 | 0,88 |
| PA_Social7 | Others appreciate me when I participate. | 3,29 | 1,35 | 0,56 | 0,88 |
| PA_Social8 | I can talk to my friends about sports. | 3,63 | 1,38 | 0,46 | 0,89 |
| PA_Social9 | I receive recognition for my performance in sports. | 3,24 | 1,38 | 0,52 | 0,89 |
| PA_Social10 | Others like me when I participate. | 3,27 | 1,37 | 0,54 | 0,89 |
| PA_Social11 | All my friends do sports. | 3,02 | 1,27 | 0,49 | 0,89 |
| PA_Coping1 | It helps me forget my daily problems. | 3,22 | 1,45 | 0,74 | 0,82 |
| PA_Coping2 | I forget my worries. | 3,29 | 1,54 | 0,59 | 0,83 |
| PA_Coping3 | It allows me to escape from the world. | 2,63 | 1,42 | 0,58 | 0,84 |
| PA_Coping4 | Sports help me feel better when I am frustrated. | 3,57 | 1,40 | 0,72 | 0,82 |
| PA_Coping5 | Doing sports improves my mood. | 3,92 | 1,10 | 0,50 | 0,84 |
| PA_Coping6 | It helps me release negative energy. | 3,55 | 1,57 | 0,68 | 0,82 |
| PA_Coping7 | I don’t feel excluded because of it. | 2,86 | 1,51 | 0,42 | 0,85 |
| PA_Coping8 | When I am angry or upset with someone, sports help me avoid a confrontation. | 2,92 | 1,50 | 0,53 | 0,84 |
| PA_Competition1 | I like defeating other athletes. | 3,12 | 1,45 | 0,61 | 0,72 |
| PA_Competition2 | I like winning. | 4,12 | 1,22 | 0,51 | 0,75 |
| PA_Competition3 | I like proving that I am better than others. | 2,90 | 1,56 | 0,73 | 0,69 |
| PA_Competition4 | I like provoking other athletes. | 1,84 | 1,26 | 0,48 | 0,76 |
| PA_Competition5 | I feel capable in sports. | 3,73 | 1,30 | 0,23 | 0,81 |
| PA_Competition6 | I enjoy competing with others. | 3,43 | 1,46 | 0,61 | 0,72 |
| PA_Skill1 | Doing sports is a mental challenge. | 3,69 | 1,08 | 0,34 | 0,75 |
| PA_Skill2 | Doing sports makes me smarter. | 3,22 | 1,34 | 0,54 | 0,70 |
| PA_Skill3 | Doing sports makes me think. | 2,94 | 1,38 | 0,53 | 0,71 |
| PA_Skill4 | Doing sports sharpens my senses. | 3,67 | 1,14 | 0,66 | 0,67 |
| PA_Skill5 | Doing sports excites me. | 3,12 | 1,41 | 0,40 | 0,75 |
| PA_Skill6 | Doing sports improves my skills. | 4,14 | 1,14 | 0,51 | 0,71 |

**Digital Gaming children**

|  | **Statement** | M | SD | rᵢ₋ₜ | α = Cronbach’s alpha if item deleted |
| --- | --- | --- | --- | --- | --- |
| G_Recreation1 | I enjoy playing video games. | 4,43 | 1,02 | 0,66 | 0,76 |
| G_Recreation2 | Video games entertain me. | 4,37 | 1,05 | 0,60 | 0,77 |
| G_Recreation3 | I have fun. | 4,73 | 0,60 | 0,67 | 0,77 |
| G_Recreation4 | It is entertaining. | 4,47 | 0,98 | 0,42 | 0,80 |
| G_Recreation5 | I like video games. | 4,71 | 0,58 | 0,38 | 0,80 |
| G_Recreation6 | It relaxes me. | 3,41 | 1,40 | 0,59 | 0,77 |
| G_Recreation7 | It helps me relieve stress. | 3,31 | 1,46 | 0,63 | 0,77 |
| G_Social1 | I make new friends. | 2,98 | 1,70 | 0,70 | 0,91 |
| G_Social2 | It allows me to meet new people. | 3,18 | 1,60 | 0,69 | 0,91 |
| G_Social3 | I stay in touch with my friends through gaming. | 3,53 | 1,49 | 0,76 | 0,91 |
| G_Social4 | I enjoy playing video games in a group. | 3,98 | 1,33 | 0,68 | 0,91 |
| G_Social5 | I like playing with others online or in the same room. | 4,12 | 1,27 | 0,53 | 0,92 |
| G_Social6 | It makes me feel part of a group I like. | 3,27 | 1,48 | 0,79 | 0,90 |
| G_Social7 | Others appreciate me when I play. | 3,29 | 1,38 | 0,81 | 0,90 |
| G_Social8 | I can talk to my friends about video games. | 3,92 | 1,43 | 0,67 | 0,91 |
| G_Social9 | I receive recognition for my gaming achievements. | 3,14 | 1,61 | 0,60 | 0,91 |
| G_Social10 | Others like me when I play. | 3,24 | 1,42 | 0,62 | 0,91 |
| G_Social11 | All my friends play video games. | 3,78 | 1,31 | 0,61 | 0,91 |
| G_Coping1 | It helps me forget my daily problems. | 3,43 | 1,34 | 0,73 | 0,83 |
| G_Coping2 | I forget my worries. | 3,10 | 1,50 | 0,65 | 0,84 |
| G_Coping3 | It allows me to escape from the real world. | 3,35 | 1,61 | 0,70 | 0,83 |
| G_Coping4 | Gaming helps me feel better when I am frustrated. | 3,27 | 1,50 | 0,75 | 0,83 |
| G_Coping5 | Gaming improves my mood. | 3,88 | 1,11 | 0,43 | 0,86 |
| G_Coping6 | It helps me release negative energy. | 3,29 | 1,34 | 0,69 | 0,84 |
| G_Coping7 | I don’t feel excluded because of it. | 2,51 | 1,57 | 0,43 | 0,87 |
| G_Coping8 | When I am angry or upset with someone, gaming helps me avoid a confrontation. | 2,98 | 1,55 | 0,52 | 0,86 |
| G_Competition1 | I like defeating other video game players. | 3,90 | 1,33 | 0,80 | 0,70 |
| G_Competition2 | I like winning. | 4,33 | 1,09 | 0,43 | 0,78 |
| G_Competition3 | I like proving that I am better than others. | 3,14 | 1,50 | 0,70 | 0,72 |
| G_Competition4 | I like provoking other video game players. | 2,63 | 1,58 | 0,47 | 0,78 |
| G_Competition5 | I feel capable in video games. | 3,41 | 1,43 | 0,30 | 0,82 |
| G_Competition6 | I enjoy competing with others. | 3,57 | 1,34 | 0,62 | 0,74 |
| G_Skill1 | Video games are a mental challenge. | 3,73 | 1,19 | 0,57 | 0,79 |
| G_Skill2 | Video games make me smarter. | 3,04 | 1,38 | 0,72 | 0,75 |
| G_Skill3 | Video games make me think. | 2,76 | 1,36 | 0,43 | 0,82 |
| G_Skill4 | Video games sharpen my senses. | 3,22 | 1,49 | 0,84 | 0,72 |
| G_Skill5 | Video games excite me. | 3,24 | 1,30 | 0,21 | 0,86 |
| G_Skill6 | Video games improve my skills. | 3,61 | 1,44 | 0,73 | 0,75 |

**Physical Activity Adults**

|  | **Statement** | M | SD | rᵢ₋ₜ | α = Cronbach’s alpha if item deleted |
| --- | --- | --- | --- | --- | --- |
| PA_Recreation1 | They enjoy doing sports. | 4,04 | 0,82 | 0,49 | 0,70 |
| PA_Recreation2 | They are entertained by doing sports. | 3,87 | 1,04 | 0,30 | 0,74 |
| PA_Recreation3 | They have fun. | 4,16 | 1,07 | 0,32 | 0,74 |
| PA_Recreation4 | It is entertaining. | 4,22 | 0,77 | 0,70 | 0,66 |
| PA_Recreation5 | They like the sport. | 4,31 | 1,02 | 0,61 | 0,66 |
| PA_Recreation6 | It relaxes them. | 3,87 | 0,99 | 0,32 | 0,73 |
| PA_Recreation7 | It helps them relieve stress. | 3,80 | 1,12 | 0,50 | 0,69 |
| PA_Social1 | They make new friends. | 3,78 | 1,15 | 0,78 | 0,91 |
| PA_Social2 | It allows them to meet new people. | 3,71 | 1,14 | 0,75 | 0,91 |
| PA_Social3 | They stay in touch with their friends through sports. | 3,82 | 1,03 | 0,69 | 0,92 |
| PA_Social4 | They enjoy doing sports in a group. | 3,78 | 1,02 | 0,81 | 0,91 |
| PA_Social5 | They like doing sports with others. | 4,02 | 0,97 | 0,70 | 0,92 |
| PA_Social6 | It makes them feel part of a group they like. | 3,76 | 1,09 | 0,60 | 0,92 |
| PA_Social7 | Others appreciate them when they participate. | 3,58 | 1,18 | 0,76 | 0,91 |
| PA_Social8 | They can talk to their friends about sports. | 3,69 | 1,00 | 0,65 | 0,92 |
| PA_Social9 | They receive recognition for their performance in sports. | 3,69 | 1,08 | 0,58 | 0,92 |
| PA_Social10 | Others like them when they participate. | 3,58 | 1,14 | 0,77 | 0,91 |
| PA_Social11 | All their friends do sports. | 3,49 | 1,14 | 0,51 | 0,92 |
| PA_Coping1 | It helps them forget their daily problems. | 3,53 | 1,01 | 0,66 | 0,70 |
| PA_Coping2 | They forget their worries. | 3,67 | 0,98 | 0,68 | 0,70 |
| PA_Coping3 | It allows them to escape from the world. | 3,00 | 1,31 | 0,51 | 0,73 |
| PA_Coping4 | Sports help them feel better when they are frustrated. | 3,87 | 0,76 | 0,44 | 0,74 |
| PA_Coping5 | Doing sports improves their mood. | 4,04 | 0,90 | 0,31 | 0,76 |
| PA_Coping6 | It helps them release negative energy. | 3,76 | 1,03 | 0,56 | 0,72 |
| PA_Coping7 | They don’t feel excluded because of it. | 3,24 | 1,13 | 0,27 | 0,77 |
| PA_Coping8 | When they are angry or upset with someone, sports help them avoid a confrontation. | 2,89 | 1,23 | 0,33 | 0,76 |
| PA_Competition1 | They like defeating other athletes. | 3,33 | 1,26 | 0,75 | 0,80 |
| PA_Competition2 | They like winning. | 3,53 | 1,36 | 0,55 | 0,85 |
| PA_Competition3 | They like proving that they are better than others. | 3,33 | 1,33 | 0,83 | 0,79 |
| PA_Competition4 | They like provoking other athletes. | 2,02 | 1,16 | 0,51 | 0,85 |
| PA_Competition5 | They feel capable in sports. | 3,93 | 0,91 | 0,37 | 0,87 |
| PA_Competition6 | They enjoy competing with others. | 3,31 | 1,29 | 0,81 | 0,79 |
| PA_Skill1 | Doing sports is a mental challenge. | 3,24 | 1,19 | 0,57 | 0,60 |
| PA_Skill2 | Doing sports makes them smarter. | 3,20 | 1,18 | 0,64 | 0,58 |
| PA_Skill3 | Doing sports makes them think. | 2,76 | 1,13 | 0,21 | 0,72 |
| PA_Skill4 | Doing sports sharpens their senses. | 3,78 | 1,00 | 0,51 | 0,63 |
| PA_Skill5 | Doing sports excites them. | 3,40 | 1,12 | 0,33 | 0,69 |
| PA_Skill6 | Doing sports improves their skills. | 4,09 | 0,76 | 0,35 | 0,68 |

**Digital Gaming Adults**

|  | **Statement** | M | SD | rᵢ₋ₜ | α = Cronbach’s alpha if item deleted |
| --- | --- | --- | --- | --- | --- |
| G_Recreation1 | They enjoy playing video games. | 4,04 | 1,07 | 0,87 | 0,82 |
| G_Recreation2 | They are entertained by video games. | 4,16 | 0,93 | 0,59 | 0,86 |
| G_Recreation3 | They have fun. | 4,49 | 0,87 | 0,78 | 0,84 |
| G_Recreation4 | It is entertaining. | 4,22 | 1,04 | 0,64 | 0,85 |
| G_Recreation5 | They like video games. | 4,42 | 0,89 | 0,80 | 0,84 |
| G_Recreation6 | It relaxes them. | 3,18 | 1,30 | 0,46 | 0,88 |
| G_Recreation7 | It helps them relieve stress. | 3,27 | 1,36 | 0,57 | 0,87 |
| G_Social1 | They make new friends. | 2,80 | 1,22 | 0,65 | 0,92 |
| G_Social2 | It allows them to meet new people. | 2,91 | 1,33 | 0,71 | 0,92 |
| G_Social3 | They stay in touch with their friends through gaming. | 3,56 | 1,24 | 0,60 | 0,92 |
| G_Social4 | They enjoy playing video games in a group. | 3,69 | 1,10 | 0,79 | 0,92 |
| G_Social5 | They like playing with others online or in the same room. | 3,64 | 1,23 | 0,70 | 0,92 |
| G_Social6 | It makes them feel part of a group they like. | 3,31 | 1,12 | 0,66 | 0,92 |
| G_Social7 | Others appreciate them when they play. | 3,31 | 1,28 | 0,80 | 0,91 |
| G_Social8 | They can talk to their friends about video games. | 3,87 | 1,18 | 0,71 | 0,92 |
| G_Social9 | They receive recognition for their gaming achievements. | 3,42 | 1,14 | 0,72 | 0,92 |
| G_Social10 | Others like them when they play. | 3,62 | 1,11 | 0,79 | 0,92 |
| G_Social11 | All their friends play video games. | 3,98 | 1,06 | 0,58 | 0,92 |
| G_Coping1 | It helps them forget their daily problems. | 3,18 | 1,27 | 0,83 | 0,86 |
| G_Coping2 | They forget their worries. | 3,38 | 1,21 | 0,75 | 0,87 |
| G_Coping3 | It allows them to escape from the real world. | 3,18 | 1,25 | 0,60 | 0,88 |
| G_Coping4 | Gaming helps them feel better when they are frustrated. | 3,07 | 1,27 | 0,76 | 0,86 |
| G_Coping5 | Gaming improves their mood. | 3,36 | 1,09 | 0,47 | 0,89 |
| G_Coping6 | It helps them release negative energy. | 3,09 | 1,28 | 0,66 | 0,87 |
| G_Coping7 | They don’t feel excluded because of it. | 3,36 | 1,25 | 0,61 | 0,88 |
| G_Coping8 | When they are angry or upset with someone, gaming helps them avoid a confrontation. | 2,91 | 1,29 | 0,60 | 0,88 |
| G_Competition1 | They enjoy defeating other video game players. | 3,67 | 1,30 | 0,81 | 0,74 |
| G_Competition2 | They like winning. | 4,09 | 1,20 | 0,43 | 0,83 |
| G_Competition3 | They like proving that they are better than others. | 3,56 | 1,14 | 0,68 | 0,78 |
| G_Competition4 | They like provoking other video game players. | 2,33 | 1,38 | 0,44 | 0,83 |
| G_Competition5 | They feel capable in video games. | 3,38 | 1,39 | 0,62 | 0,79 |
| G_Competition6 | They enjoy competing with others. | 3,71 | 1,16 | 0,60 | 0,79 |
| G_Skill1 | Video games are a mental challenge. | 3,36 | 1,13 | 0,47 | 0,71 |
| G_Skill2 | Video games make them smarter. | 2,56 | 1,12 | 0,58 | 0,68 |
| G_Skill3 | Video games make them think. | 2,69 | 1,22 | 0,52 | 0,69 |
| G_Skill4 | Video games sharpen their senses. | 3,40 | 1,23 | 0,63 | 0,66 |
| G_Skill5 | Video games excite them. | 3,69 | 1,12 | 0,07 | 0,81 |
| G_Skill6 | Video games improve their skills. | 3,42 | 1,14 | 0,65 | 0,66 |
